# Kampo Medicine: Evaluation of the Pharmacological Activity of 121 Herbal Drugs on GABA_A_ and 5-HT_3A_ Receptors

**DOI:** 10.3389/fphar.2016.00219

**Published:** 2016-07-29

**Authors:** Katrin M. Hoffmann, Robin Herbrechter, Paul M. Ziemba, Peter Lepke, Leopoldo Beltrán, Hanns Hatt, Markus Werner, Günter Gisselmann

**Affiliations:** ^1^Department of Cell Physiology, Ruhr University BochumBochum, Germany; ^2^Kronen Apotheke WuppertalWuppertal, Germany

**Keywords:** Kampo, *Leonurus japonicus* (herba), *Panax ginseng* (radix), *Salvia miltiorrhiza* (radix), andrographolide, leonurine, 5-HT_3A_ receptor, GABA_A_ receptor

## Abstract

Kampo medicine is a form of Japanese phytotherapy originating from traditional Chinese medicine (TCM). During the last several decades, much attention has been paid to the pharmacological effects of these medical plants and their constituents. However, in many cases, a systematic screening of Kampo remedies to determine pharmacologically relevant targets is still lacking. In this study, a broad screening of Kampo remedies was performed to look for pharmacologically relevant 5-HT_3A_ and GABA_A_ receptor ligands. Several of the Kampo remedies are currently used for symptoms such as nausea, emesis, gastrointestinal motility disorders, anxiety, restlessness, or insomnia. Therefore, the pharmacological effects of 121 herbal drugs from Kampo medicine were analyzed as ethanol tinctures on heterologously expressed 5-HT_3A_ and GABA_A_ receptors, due to the involvement of these receptors in such pathophysiological processes. The tinctures of *Lindera aggregata* (radix) and *Leonurus japonicus* (herba) were the most effective inhibitory compounds on the 5-HT_3A_ receptor. Further investigation of known ingredients in these compounds led to the identification of leonurine from *Leonurus* as a new natural 5-HT_3A_ receptor antagonist. Several potentiating herbs (e.g., *Magnolia officinalis* (cortex), *Syzygium aromaticum* (flos), and *Panax ginseng* (radix)) were also identified for the GABA_A_ receptor, which are all traditionally used for their sedative or anxiolytic effects. A variety of tinctures with antagonistic effects *Salvia miltiorrhiza* (radix) were also detected. Therefore, this study reveals new insights into the pharmacological action of a broad spectrum of herbal drugs from Kampo, allowing for a better understanding of their physiological effects and clinical applications.

## Introduction

The 5-HT_3A_ and GABA_A_ receptors are ionotropic receptors within the cys-loop superfamily of ligand-gated ion channels and therefore possess closely related structures. Both of these receptors share a pentameric structure, with each subunit consisting of four transmembrane domains (Connolly and Wafford, 2004). The physiological agonists of the receptors are gamma aminobutyric acid (GABA) and serotonin (5-HT), respectively. There are many pharmacological similarities between the 5-HT_3_ and GABA_A_ receptors. For example, the plant-derived compound picrotoxin acts as a non-competitive antagonist of both receptors (Das and Dillon, 2005), and local anesthetics, such as lidocaine and procaine, can antagonize either receptor (Hara and Sata, 2007; Ueta et al., 2007). A number of GABA_A_ receptor potentiators or agonists, such as propofol, methohexital, and pentobarbital, are capable of antagonizing the 5-HT_3_ receptor (Olsen et al., 1991; Cestari et al., 1996; Barann et al., 2000, 2008). However, while the 5-HT_3_ receptor displays a cationic selectivity, activation of the GABA_A_ receptor triggers an influx of Cl^-^, resulting in the hyperpolarization of the cell and reduced neuronal excitability. Because of this, the activation of GABA_A_ receptors leads to sedative effects. Therefore, GABA_A_ receptor potentiators and agonists are broadly used for restlessness and insomnia (Calcaterra and Barrow, 2014). For example, the allosteric GABA_A_ receptor potentiator diazepam is commonly used for psychiatric disorders, including anxiety and epilepsy (Calcaterra and Barrow, 2014). The 5-HT_3_ receptors are also involved in many pathophysiological processes, such as gastrointestinal motility disorders and the development of nausea and vomiting. Therefore, compounds that act on this receptor have broad clinical relevance (Doak and Sawynok, 1997; Gershon, 2004; Jeggo et al., 2005; Costedio et al., 2007). Specific 5-HT_3_ receptor antagonists such as ondansetron are mainly used for the treatment of nausea for various conditions, including chemotherapy-induced nausea and vomiting (CINV) and nausea during the postoperative phase (PONV; Cubeddu et al., 1994; Gyermek, 1995).

Kampo is a form of traditional Japanese phytomedicine originating from traditional Chinese medicine (TCM). Typically, Kampo is administered as a mixture of various herbal drugs that have complementary physiological activities. Kampo is broadly used in alternative and complementary medicine and has also recently become popular in Western countries. Therefore, there is a general interest in understanding the underlying pharmacological mechanisms of these herbal drugs, which may also help to increase the impact of Kampo in Western medicine. Several previous reports have described the pharmacological actions of specific components of single plants (e.g., gingerol from *Zingiber officinalis*) or complex Kampo preparations consisting of multiple components, such as rikkunshito (Takeda et al., 2008; Tominaga et al., 2011; Herbrechter et al., 2015), on pharmacologically relevant targets, including G-protein-coupled receptors (GPCRs) and ion channels. During the last decades, screening of the pharmacological activity of plant extracts and subsequent trials to identify their active ingredients has led to a plethora of pharmacologically useful substances. Kampo medicine depends on a relatively limited number of 148 well-described mostly plant-derived ingredients (Watanabe et al., 2011). However, only a few attempts were made to establish a systematic and comprehensive screening of the action of all of the important Kampo or TCM preparations on specific drug targets, such as ion channels. Nevertheless, such investigations have led to the identification of new pharmacological tools, as demonstrated by the screening of 50 Chinese herbal plants, which led to the identification of bisandrographolide as the first natural TRPV4 activator (Smith et al., 2006).

Several of the Kampo remedies are used to treat symptoms such as nausea, emesis, gastrointestinal motility disorders, anxiety, restlessness, and insomnia. Therefore, the pharmacological effects of 121 herbal compounds from Kampo medicine were analyzed as ethanol tinctures on the heterologously expressed 5-HT_3A_ and GABA_A_ receptors, due to their involvement in the above pathophysiological processes. We aimed to investigate if there is a correlation between the pharmacological action of the Kampo compounds on these receptors and the corresponding medical application. We further sought to establish an activity ranking and identify the most potent tinctures as well as new active ingredients.

## Materials and Methods

### Tinctures and Substances

The ethanol tinctures of 121 Kampo remedies were obtained from Dr. Peter Lepke (Kronen Apotheke Wuppertal, Germany). Tinctures were made by extracting the plant material in ethanol (1:5 w/v). The tinctures used in this study are listed in **Supplementary Table S1**. All of the chemicals were purchased from either Sigma–Aldrich (5-HT hydrochloride, gamma-aminobutyric acid, tannic acid, schizandrin, schizandrin B, leonurine, boldine, berberine chloride, liquiritigenin, hesperetin, kaempferol, andrographolide, linderane, rosmarinic acid, 4-hydroxybenzaldehyd, chlorogenic acid, caffeic acid), Carl Roth (rutin) or PhytoLab (atractylenolide III).

### Expression System

The expression plasmid pRc/CMV contained cDNA coding for the human 5-HT_3A_ protein (Invitrogen; Lankiewicz et al., 1998), and psGEM contained cDNA for the α1, β2, and γ2 GABA_A_ receptor subunits (Sergeeva et al., 2010). cRNAs were prepared using the AmpliCap T7 high-yield message marker kit (Epicenter, Madison, WI, USA), following the manufacturer’s protocol. Oocytes were obtained as previously described (Sherkheli et al., 2010), and 7–20 ng receptor coding cRNA was injected into the oocytes using an injection setup from WPI (Nanoliter 2000, Micro4). The injected oocytes were then stored in ND 96 (96.0 mM NaCl, 2.0 mM KCl, 1.8 mM CaCl_2_, 1.0 mM MgCl_2_, 5.0 mM HEPES, pH 7.2, 200 U/ml penicillin, 200 μg/ml streptomycin) at 14°C. Electrophysiological experiments were performed 1–5 days (5-HT_3A_) or 2–3 days (GABA_A_) after the cRNA-injections.

### Electrophysiology

To investigate the heterologously expressed 5-HT_3A_ and GABA_A_ receptors, the two-electrode voltage-clamp technique was used as described previously (Saras et al., 2008). All of the recordings were performed in a normal frog ringer’s (NFR) buffer (115 mM NaCl, 2.5 mM KCl, 1.8 mM CaCl_2_, 10 mM HEPES, pH 7.2) for the GABA_A_ receptor. Currents were recorded at a holding potential of approximately -60 mV (5-HT_3A_ receptor) or -40 mV (GABA_A_ receptors), using the software Cell Works 6.1.1. (NPI). During the measurements, the oocytes were placed in a chamber with a constant and unidirectional ringer flow, allowing the washout of the tested substances and tinctures. The ringer flow was interrupted for the application of test substances and tinctures until the maximal response was transcended. To exclude solvent effects, the compounds were tested at the maximal applied concentration (0.1 Vol.-%). Neither ethanol nor DMSO exhibited a direct activation or modulatory effect on the 5-HT_3A_ and GABA_A_ receptors at the examined concentration (data not shown). In addition, the action of the tinctures on non-injected oocytes was tested (1:1,000 dilution; *n* = 3). None of the tinctures exhibited a direct activating effect with currents >15 nA (**Supplementary Table S2**). The observed effects were negligible compared with typical agonist-induced currents (∼2 μA). The direct activating effects of the Kampo tinctures were examined using a 1:1,000 dilution. The diluted tinctures were applied to *Xenopus laevis* oocytes expressing either the 5-HT_3A_ or α1β2γ2 GABA_A_ receptor and compared the evoked responses with the response from 5 μM 5-HT or 100 μM GABA, respectively (*n* = 3–8). For the investigation of the modulatory effects, the tinctures were applied in a 1:1,000-dilutuion witch contains the native agonists (*n* = 3–8). Concentrations of 10 μM GABA for the GABA_A_ receptor and 5 μM 5-HT for the 5-HT_3A_ receptor were used. To identify the active compounds in tinctures with antagonistic and potentiating effects, the known ingredients of plants with previously identified potential bioactivity were tested at a concentration of 1 mM. This approach was successfully used to identify pharmacologically active plant ingredients in a previous study (Herbrechter et al., 2015).

### Data Analysis

The currents of the tested substances/tinctures were normalized to the means of the reference compounds 5-HT and GABA for the 5-HT_3A_ and α1β2γ2 GABA_A_ receptors, respectively. Sigmoidal regression analysis was performed using the 3- or 4-parameter Hill equation (SigmaPlot 8.0., SPSS) to fit concentration-effect curves and calculate the IC/EC_50_ values. Deviations are represented by the standard error of the mean (SEM). Significant differences were determined using Student’s *t*-test (Excel 2010, Microsoft), and multiple comparisons were corrected using the Benjamini–Hochberg-correction (^∗^*p* < 0.05; ^∗∗^*p* < 0.005; ^∗∗∗^*p* < 0.0005).

## Results

### The Effect of Kampo Tinctures on the 5-HT_3A_ and GABA_A_ Receptors

**FIGURE 1:**
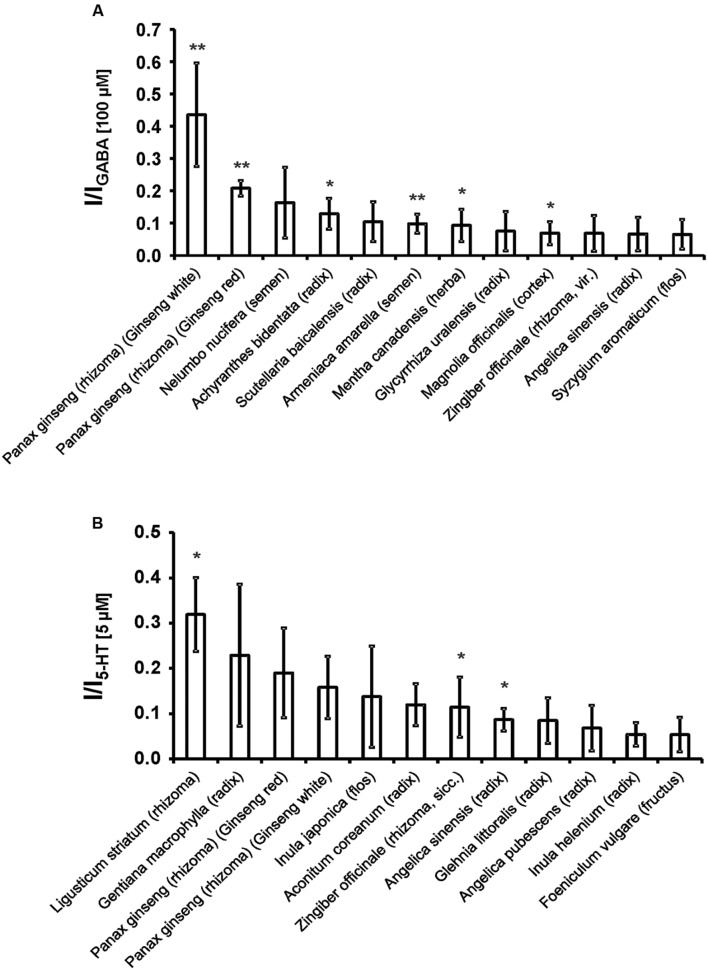
**The strongest 12 direct activating tinctures for the 5-HT_3A_**(A)** and GABA_A_ receptors **(B)**.** The 121 tinctures were made from Kampo remedies via ethanol extraction (see section Tinctures and substances). A 1:1,000-dilution was applied to the oocytes and compared with agonist induced currents (5 μM 5-HT, 100 μM GABA). Error bars represent the SEM. Statistical significance was calculated based on the current evoked by ethanol (0.1 Vol.-%; ^∗^*p* < 0.05, ^∗∗^*p* < 0.005; *n* = 3–5).

First, the direct activating effects of the tinctures were investigated. The tincture of *Ligusticum striatum* (rhizoma) showed the strongest activation of the 5-HT_3A_ receptor, with more than 30% of the 5-HT-induced current (**Figure 1**; **Supplementary Table S2**). The strongest activator of the GABA_A_ receptor was *Panax ginseng* (rhizoma; Ginseng white), with a mean current amplitude of more than 40% of the GABA-induced current. The strongest 12 direct activating tinctures are shown in **Figure 1.**

**FIGURE 2:**
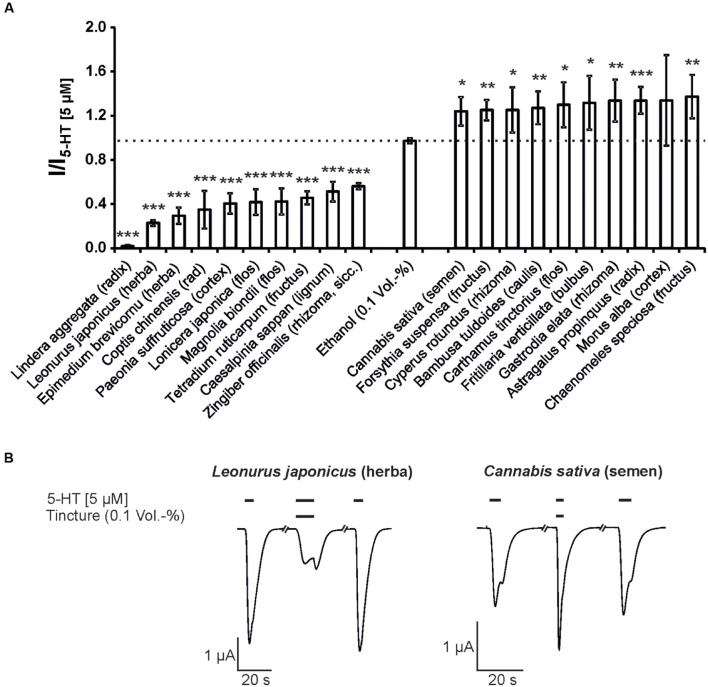
**The modulatory action of the strongest inhibitory and potentiating Kampo tinctures on the 5-HT_3A_ receptor.** **(A)** The tinctures with the 10 strongest antagonistic (left) and potentiating (right) effects are shown (5 μM 5-HT). Bars exceeding the dotted line represent potentiating effects. Statistical significance was calculated based on the reference solution containing 0.1 Vol.-% ethanol (equal to the max. ethanol concentration in the tinctures; ^∗^*p* < 0.05, ^∗∗^*p* < 0.005, ^∗∗∗^*p* < 0.0005; *n* = 3–8). **(B)** Original registrations of the modulation observed by *Leonurus japonicus* (herba) and *Cannabis sativa* (semen) using a 1:1,000 dilution are shown. The effects were reversible following a 150-s washout with ringer’s solution.

**FIGURE 3:**
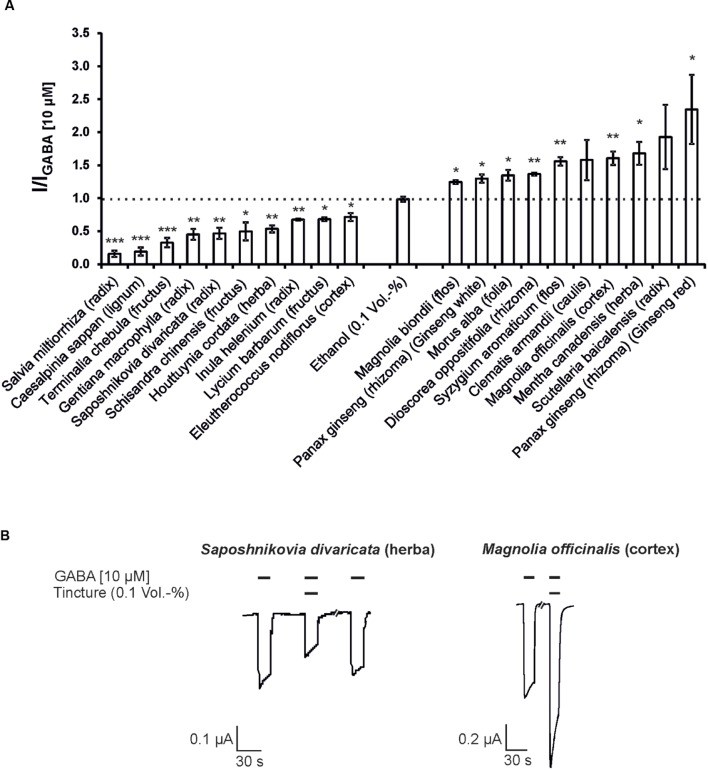
**The modulatory action of the strongest inhibitory and potentiating Kampo tinctures on the GABA_A_ receptors.** **(A)** The tinctures with the 10 strongest antagonistic (left) and potentiating (right) effects are shown (10 μM GABA). Statistical significance was calculated based on the reference solution containing 0.1 Vol.-% ethanol (equal to the max. ethanol concentration in the tinctures; ^∗^*p* < 0.05, ^∗∗^*p* < 0.005, ^∗∗∗^*p* < 0.0005; *n* = 3–8). **(B)** Original registrations of the modulation observed via *Saposhnikovia divaricata* (herba) and *Magnolia officinalis* (cortex) with a 1:1,000 dilution are shown. The effects were reversible following a 150-s washout with ringer’s solution.

Next, the modulatory effects of the tinctures at a 1:1,000 dilution was evaluated. In these experiments, agonist concentrations of 10 μM GABA for the GABA_A_ receptor and 5 μM 5-HT for the 5-HT_3A_ receptor were used. A variety of tinctures with modulatory action for both receptor types were identified. For the 5-HT_3A_ receptor, tinctures with significant antagonistic and potentiating effects were identified (13 tinctures with potentiating effects and 22 tinctures with inhibitory potential). The strongest potentiation was observed with *Chaenomeles speciosa* (fructus), which potentiated the 5-HT-induced response with only 37% (**Figure 2**). In contrast, eight tinctures [*Tetradium ruticarpum* (fructus), *Magnolia biondii* (flos), *Lonicera japonica* (caulis), *Paeonia × suffruticosa* (cortex), *Coptis chinensis* (radix), *Epimedium brevicornum* (herba), *Leonurus japonicus* (herba), and *Lindera aggregata* (radix)) exceeded 50% inhibition of the receptor, with a maximal inhibition of more than 98% by the *Lindera aggregata* (radix) tincture. In addition, the GABA_A_ receptors were significantly potentiated by 14 tinctures and inhibited by 24 tinctures. The *Syzygium aromaticum* (flos), *Clematis armandii* (caulis), *Magnolia officinalis* (cortex), *Mentha canadensis* (herba), *Scutellaria baicalensis* (radix), and *Panax ginseng* (rhizoma; Ginseng red) tinctures exhibited the strongest potentiation. In particular, Ginseng red potentiated up to 135% (**Figure 3**). The most effective inhibitory tinctures were *Salvia miltiorrhiza* (radix), *Caesalpinia sappan* (lignum), *Terminalia chebula* (fructus), *Gentiana macrophylla* (radix), *Saposhnikovia divaricata* (radix), and *Schisandra chinensis* (fructus). The inhibition observed by the *Salvia miltiorrhiza* (radix) tincture exceeded an inhibition of 80% of the GABA-induced currents.

### Investigation of Established Ingredients in the Active Kampo Tinctures

For several of the active tinctures, the active ingredients have already been identified. However, for many of the Kampo herbs that have been shown to act on the 5-HT_3A_ or GABA_A_ receptor, the active ingredients are unknown. The investigated substances and their related plants are listed in **Table 1** and **Supplementary Table S3**.

**Table 1:** Investigated ingredients and their respective plants.

Substance	Kampo remedy	Reference
4-hydroxybenzaldehyd	*Lonicera japonica* (caulis)	Wang et al., 2013
andrographolide	*Andrographis paniculata* (herba)	Song et al., 2013
Atractylenolid III	*Atractylodes lancea* (rhizoma)	Shao et al., 2014
	*Atractylodis macrocephala* (rhizoma)	
Berberine	*Coptis chinensis* (radix)	Lin et al., 2004
Boldine	*Lindera aggregata* (radix)	Han et al., 2008
Chlorogenic acid	*Inula helenium* (radix)	Eberhard, 2003;
	*Lonicera japonica* (caulis)	Wang et al., 2014
Caffeic acid	*Inula helenium* (radix)	Wang et al., 2014
Hesperetin	*Citrus × aurantium* (fructus)	Zhao et al., 2015
	*Citrus trifoliata* (fructus)	
	*Citrus reticulata* (*pericarpium, vir.*)	
	*Citrus reticulata* (pericarpium)	
Kaempferol	*Houttuynia cordata* (herba)	Lin et al., 2013
Leonurine	*Leonurus japonicus* (herba)	Chen and Kwan, 2001
Linderane	*Lindera aggregata* (radix)	Li et al., 2002
Liquiritigenin	*Glycyrrhiza uralensis* (radix)	Rauchensteiner et al., 2005; Kondo et al., 2007
Rosmarinic acid	*Salvia miltiorrhiza* (radix)	Adams et al., 2006; Huang et al., 2008
Rutin	*Citrus × aurantium* (fructus)	Hunyadi et al., 2012; Zhao et al., 2015
	*Citrus trifoliata* (fructus)	
	*Citrus reticulata* (*pericarpium, vir.*)	
	*Citrus reticulata* (pericarpium)	
	*Morus alba* (folia)	
Schizandrin B	*Schisandra chinensis* (fructus)	Pan et al., 2008
Schizandrin	*Schisandra chinensis* (fructus)	Panossian and Wikman, 2010
Tannic acid	*Syzygium aromaticum* (flos)	Bhowmik et al., 2012


**FIGURE 4:**
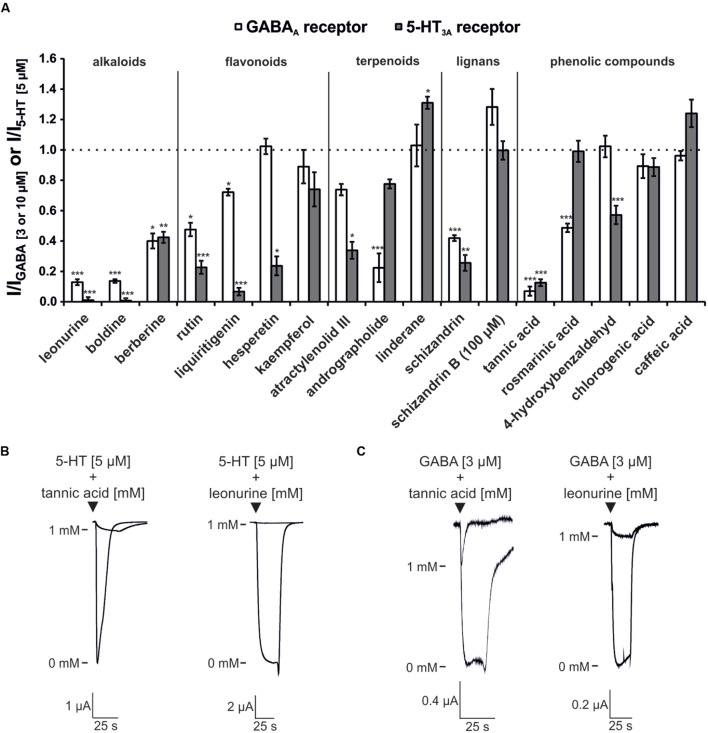
**Modulatory effects of the investigated ingredients on the 5-HT_3A_ and GABA_A_ receptors.** **(A)** The bars represent the normalized current amplitude for the modulation of the 5-HT_3A_ (gray bars) and GABA_A_ (white bars) receptors. With the exception of schizandrin B, which was tested at 100 μM, all of the compounds were evaluated at a concentration of 1 mM. Bars exceeding the dotted line represent substances with potentiating effects. The substances are arranged based on their affiliation to classes of plant metabolites, as indicated by the vertical lines. Statistical differences were calculated based on the reference agonist, which contained an equal concentration of the solvent used to dissolve the test compounds (^∗^*p* < 0.05, ^∗∗^*p* < 0.005, ^∗∗∗^*p* < 0.0005; *n* = 4–7). **(B)** Original registrations of the inhibition of the 5-HT_3A_ receptor by tannic acid and leonurine. **(C)** Original registrations of the inhibition of the GABA_A_ receptor by tannic acid and leonurine.

The modulatory effects of these substances on both receptors are shown in **Figure 4.** Overall, alkaloid compounds were found to be the most effective antagonists. In particular, boldine [*Lindera aggregata* (radix)] and leonurine [*Leonurus japonicus* (herba)] each possessed a higher efficacy for the 5-HT_3A_ receptor than the GABA_A_ receptor. These compounds were the most potent 5-HT_3A_ receptor antagonists in the screening (**Figure 4**). The alkaloid berberine inhibited both receptor types at approximately 60%. Another identified 5-HT_3A_ receptor antagonist is the flavonoid (-)-liquiritigenin (Herbrechter et al., 2015), which showed nearly no effect on GABA_A_ receptors in this screen. All of the tested flavonoids inhibited 5-HT_3A_ receptors with higher efficacy than GABA_A_ receptors. However, many of the substances showed inhibitory potential rather than potentiating effects on the receptors, with the exception of schizandrin B, which slightly potentiated the GABA_A_ receptor. Within the phenolic compound category, tannic acid was identified as an antagonist of both receptor types, with an inhibition rate of more than 80%. Andrographolide and the lignane schizandrin were identified as weak GABA_A_ receptor antagonists.

### Concentration-Effect Curves of the Identified Ingredients with Potential Pharmaceutical Action

**FIGURE 5:**
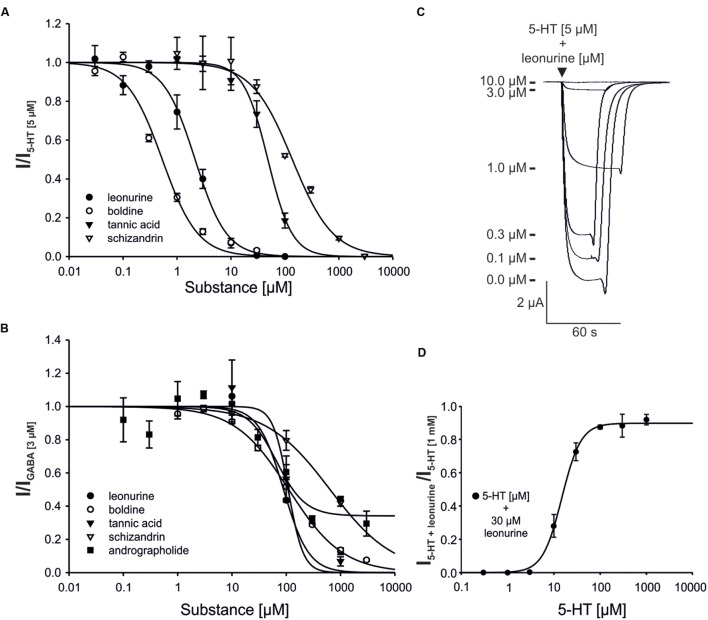
**Concentration-inhibition curves of select plant ingredients for the 5-HT_3A_**(A)** and GABA_A_**(B)** receptor, as well as the original registrations of leonurine for the 5-HT_3A_ receptor **(C)** and the 5-HT concentration-effect curve in the presence of leonurine **(D)**.** **(A,B)** The agonist concentrations used in the experiment were 5 μM (5-HT) or 3 μM (GABA). Error bars represent the SEM (**A**: *n* = 5–7, **B**: *n* = 4–7). Andrographolide acts as a partial antagonist of the GABA_A_ receptor. **(C)** Overlayed 5-HT_3A_ receptor responses, modulated by different concentrations of leonurine (0.1–10 μM) are shown. **(D)** The concentration-effect curve of 5-HT at the 5-HT_3A_ receptor in the presence of leonurine (30 μM) is shown. The determined EC_50_ value was found to be 14.8 μM 5-HT (*n* = 4).

To quantify the antagonism of the identified active ingredients, concentration-effect curves were generated (**Figure 5**; **Supplementary Table S4**).

For the 5-HT_3A_ receptor, boldine (IC_50_ = 0.53 ± 0.15 μM) and leonurine (IC_50_ = 2.17 ± 0.15 μM) were more potent inhibitors than tannic acid (IC_50_ = 48.2 ± 4.1 μM) and schizandrin (IC_50_ = 137 ± 22 μM). These four substances had approximately equal IC_50_ values for the GABA_A_ receptor (∼100 μM, **Supplementary Table S4**), with the exception of schizandrin (IC_50_ > 600 μM). Andrographolide (IC_50_ = 66.1 ± 26.8 μM) was found to be the most potent GABA_A_ receptor antagonist. However, it also acted as a partial antagonist of the GABA_A_ receptor, as even a concentration of 3 mM reduced the GABA-evoked current to 30% of the GABA reference (**Figure 5B**). Leonurine shifted the EC_50_ for 5-HT at the 5-HT_3A_ receptor from 2.5 μM determined in parallel under the same experimental conditions in our laboratory (Herbrechter et al., 2015) to 14.8 μM. While 30 μM leonurine almost completely blocked the evoked current at lower agonist concentrations, it was nearly ineffective at 5-HT concentrations ≥100 μM, suggesting a competitive antagonism of leonurine at the 5-HT_3A_ receptors.

## Discussion

In this screening, two classical drug targets, the 5-HT_3_ and GABA_A_ receptors, were investigated. Several Kampo remedies have anxiolytic, sedative, antiemetic, or digestive effects, suggesting that these receptors may be the pharmacological targets of Kampo compounds. Therefore, 121 Kampo remedies were screened as ethanol extracts on heterologously expressed α1β2γ2 GABA_A_ and homomeric 5-HT_3A_ receptors, using the two-electrode voltage-clamp technique. As a result, several remedies that can modulate the 5-HT- or GABA-induced currents were found. An activity ranking of the activity of each plant preparation on these receptors was established. By testing single substances that are present in the active plant extracts, several new 5-HT_3A_ blockers and GABA_A_ receptor modulators were identified.

### Pharmacologically Active Kampo Remedies: 5-HT_3A_ Receptor

During the screening for compounds that are pharmacologically active at the 5-HT_3A_ receptor, a number of remedies with antagonistic properties were identified. Several of these remedies, such as ginger [*Zingiber officinalis* (rhizoma)], have well-described antiemetic effects and are used for the treatment of nausea and gastrointestinal disorders (Ernst and Pittler, 2000; Haniadka et al., 2012; Ding et al., 2013). During our screen, rhizoma from *Zingiber off. sicc.* and *Zingiber off. vir.* exhibited antagonistic effects, and the effect of *Zingiber off. sicc.* (desiccated) was superior to that observed with the tincture of fresh rhizoma from *Zingiber off. vir*. This increased inhibition may be due to the manufacturing process of the tinctures, as a higher amount of dry matter was used for the *Zingiber off. sicc.* tincture, and this may have resulted in an increase in the concentration of the active ingredients within the tincture. Vanilloids from the gingerol and shogaol group of compounds, pungent substances of ginger, are well-known 5-HT_3A_ receptor antagonists. This may explain, in part, the observed inhibition of 5-HT-induced currents that have been reported previously (Walstab et al., 2013; Ziemba et al., 2015). In addition, terpenes within the essential oil of ginger, including geraniol, citronellol, linalool and galanolactone, were shown to antagonize the 5-HT_3A_ receptor (Huang et al., 1991; Ziemba et al., 2015). The antagonistic potential of *Panax ginseng* (radix) tinctures may be mediated by ginsenosides, which act in the pore region of the 5-HT_3A_ receptor, and its antiemetic properties have been previously described (Min et al., 2003; Kim et al., 2005; Lee et al., 2007). The *Magnolia biondii* (flos), *Mentha canadensis* (herba), *Glycyrrhiza uralensis* (radix) and *Syzygium aromaticum* (flos) tinctures, which are also reported to have antiemetic effects (World Health Organization, 2005; Bhowmik et al., 2012; Herbrechter et al., 2015), were among the top 20 inhibitory tinctures in the screening. *Syzygium aromaticum* (flos) contains the phenolic compounds eugenol, gallic acid, and tannins, as well as the flavonoid quercetin (Atawodi et al., 2011; Bhowmik et al., 2012). Instead of eugenol (the main flavoring ingredient in *Syzygium aromaticum*), which shows a negligible level of antagonism (Ziemba et al., 2015), tannic acid inhibits 5-HT-evoked currents with an IC_50_ value of approximately 50 μM (**Figure 5**). In addition to tannic acid, quercetin may contribute to the antagonistic effect of the clove tincture (Lee et al., 2008). *Glycyrrhiza uralensis* (radix) contains a structurally related flavonoid, liquiritigenin, which has been shown to antagonize the 5-HT_3A_ receptor in a previous study. This finding may help us to understand the antagonistic effect of this tincture (Herbrechter et al., 2015). Another flavonoid from licorice, named glabridin, was identified as a partial antagonist of the 5-HT_3A_-receptor and a potentiator of GABA_A_-receptors (Herbrechter et al., 2015; Hoffmann et al., 2016). The antagonistic effect of *Mentha canadensis* (herba) may be explained through the action of terpenes, which are known to inhibit the 5-HT_3A_ receptor. Menthol, a terpene from the essential oil of *Mentha canadensis* (herba), was recently characterized as a 5-HT_3_ receptor antagonist (Walstab et al., 2014). In addition, *Coptis chinensis* rhizomes (Tjong et al., 2011) and *Lonicera japonica* (flos; Jung et al., 2014) may be useful for the treatment of gastrointestinal disorders through the action 5-HT_3_ receptors, as discussed below.

**FIGURE 6:**
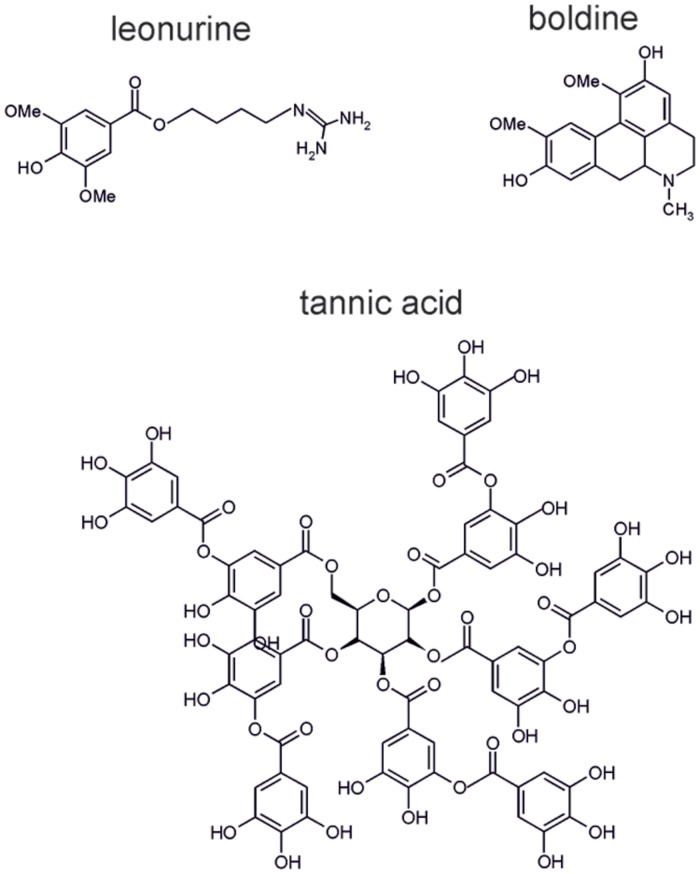
**Molecular structures of the alkaloids leonurine, boldine, and tannic acid**.

The most potent inhibitory effects were observed for the tinctures of *Lindera aggregata* (radix) and *Leonurus japonicus* (herba; **Figure 2**; **Supplementary Table S2**) for which no connection to gastrointestinal disorders is described in the literature. By screening known constituents of these plants, boldine (*Lindera*) and leonurine (*Leonurus*) are found as potent inhibitors of the 5-HT_3A_ receptor with an IC_50_ between 0.5 and 2.3 μM (**Figures 5** and **6**). Boldine, which has been shown to ameliorate chemotherapy-induced nausea and vomiting as well as symptoms from irritable bowel syndrome, is an alkaloid from the aporphine class. Boldine has been previously shown to antagonize the 5-HT_3A_ receptor in a competitive manner in a luminescence-based cell assay (Walstab et al., 2014). The contribution of further ingredients, such as the related alkaloids norboldine, reticuline, and linderegatine, to the antagonistic effect of the *Lindera* tincture is likely (Han et al., 2008). Leonurine from *Leonurus* was shown to affect cardiac function via creatine kinase inhibition (Wang et al., 2004) and to also reduce platelet aggregation (Lin et al., 2007). These effects are in accordance with some of the traditional uses of *Leonurus*. The antagonistic effects of *Leonurus* and the identification of leonurine as a potent competitive 5-HT_3A_ receptor antagonist are reported here for the first time. Therefore, this study reveals new insights into the pharmacological action of *Leonurus*. However, the traditional application of *Leonurus* is not correlated with the pharmacology of the 5-HT_3_ receptor (Shang et al., 2014). Therefore, further studies examining if *Leonurus* can antagonize the 5-HT_3A_ receptor *in vivo* are needed.

### Pharmacologically Relevant Kampo Remedies: GABA_A_ Receptor

The GABA_A_ receptors are inhibitory receptors and therefore reduce neuronal excitability. A variety of disorders are attributable to an imbalance of the GABAergic system. Therefore, benzodiazepines, such as diazepam, potentiate the GABA-induced responses and are used to treat disorders such as insomnia, restlessness, anxiety, and epilepsy (Calcaterra and Barrow, 2014).

In this screening, the potentiating activity of the tinctures correlated well with the medical use of the plants. For many of the top 10 potentiation tinctures, the active ingredients had been previously identified. The best potentiator, *Panax ginseng* (rhizoma; Ginseng red) is likely one of the most popular herbal drugs. It is traditionally used for multiple purposes, including the treatment of anxiety and insomnia (Kim et al., 2005; Xiang et al., 2008; Lee et al., 2013). The ginsenosides (steroid glycosides) present in this compound may be responsible for the potentiation and hence the sedative and anxiolytic effects of ginseng, due to their potentiating effect on the GABA_A_ receptor. The ginsenosides Rg_3_ and Rc have been shown to potentiate the heterologously expressed GABA_A_ receptor, and ginsenoside Rc also possesses agonistic properties toward the GABA_A_ receptor (Choi et al., 2003; Lee et al., 2013). This may explain the observed direct activating effect of the extract (**Figure 1**). Two *Panax ginseng* (radix) tinctures were also tested, which differ in their manufacturing process. Whereas Ginseng red is desiccated directly after harvesting, Ginseng white is peeled and whitened prior to the desiccation process. Despite these manufacturing differences, both compounds were of the best nine potentiating tinctures. However, Ginseng red is superior to Ginseng white due to its increased potentiation (135% instead of 30%; **Figure 3**; **Supplementary Table S2**).

The *Scutellaria baicalensis* (radix) tincture potentiated the GABA_A_ receptor at a similar level to *Panax ginseng* (rhizoma; Ginseng red; **Figure 3**; **Supplementary Table S2**), and it was also previously linked to GABA_A_ receptor-related effects. *Scutellaria baicalensis* (radix) possesses anticonvulsant, sedative and anxiolytic effects, which may be explained by the action of the flavone wogonin (Hui et al., 2002; Park et al., 2007). Wogonin was identified as a ligand for the benzodiazepine site of the GABA_A_ receptor and was shown to potentiate GABA_A_ receptors in electrophysiological assays (Hui et al., 2002). The observed anxiolytic effects of wogonin in mice were similar to that of diazepam, emphasizing the potency of wogonin (Hui et al., 2002). The *Mentha canadensis* (herba) and *Magnolia officinalis* (cortex) tinctures also exhibited a potentiating effect in the screening (**Figure 3**; **Supplementary Table S2**). The active components of *Magnolia officinalis* (cortex) are the lignans magnolol and its isomer honokiol, whose potentiation of the GABA_A_ receptor is accountable for the anticonvulsant, antidepressive, and anxiolytic effects of Magnolia bark (Taferner et al., 2011; Alexeev et al., 2012). The potentiation of the GABA_A_ receptor by the *Mentha* tincture likely occurs due to the action of menthol and the structurally related monoterpenoids, which were shown to potentiate GABA_A_ receptors in a previous study (Hall et al., 2004). In addition, extracts of *Morus alba* displayed anxiolytic effects under different experimental paradigms, suggesting that this compound may also act on the GABA_A_ receptor (Yadav et al., 2008). Our results support this idea, as *Morus alba* (folia) extracts belonged to the top 10 most potent GABA_A_ potentiators. However, the active component in *Morus alba* (folia) extract is still unknown.

The sixth-strongest potentiation was observed by the clove tincture [*Syzygium aromaticum* (flos)], which has anti-inflammatory, antimicrobial, antioxidant, and anesthetic effects (Chaieb et al., 2007). *In vivo*, eugenol, the main ingredient of the clove tincture, exhibits a sedative effect in mice and rats (Guenette et al., 2006; Sharma et al., 2012) and has also been shown to potentiate the effects of the GABA_A_ receptor *in vitro* (Aoshima and Hamamoto, 1999). Other potentiators were also identified, including carvacrol and thymol (Priestley et al., 2003; Kessler et al., 2014). In addition to eugenol, *Syzygium aromaticum* (flos) contains carvacrol and thymol (Priestley et al., 2003; Kessler et al., 2014), and all three are potentiators of the GABA_A_ receptor. The mutual action of these compounds may explain the sedative effects of *Syzygium aromaticum* (flos).

Antagonism of the GABA_A_ receptor is typically accompanied by stimulating effects at low doses but can cause seizures and anxiety at higher concentrations. Kampo remedies with antagonistic effects were detected, including the tinctures of *Salvia miltiorrhiza* (radix), *Terminalia chebula* (fructus), and *Schisandra chinensis* (fructus; **Figure 3**; **Supplementary Table S2**). However, we could not find an anxiogenic or seizure-inducing adverse side effect for these antagonistic tinctures in the literature. The *Schisandra chinensis* (fructus) tincture, which inhibited 50% of the GABA-induced current at a 1:1,000 dilution (**Figure 3**; **Supplementary Table S2**), possesses side effects such as restlessness and tension, when given at high doses (Eberhard, 2003). These effects may be explained, at least in part, by its antagonism of the GABA receptor. Nevertheless, *Schisandra chinensis* (fructus) is traditionally used for its hepatoprotective and sedative effects; the latter is based on the influence of the GABAergic and serotonergic systems, as the levels of their respective neurotransmitters can be altered in the brain (Ip et al., 1996; Wei et al., 2013; Zhang et al., 2014). The lignanoid schizandrin was identified as a weak inhibitor of the GABA_A_ receptor (**Figures 4** and **5**; **Supplementary Table S4**), presumably acting in a non-competitive manner (**Supplementary Figure S1**). However, its contribution to the effect of the tincture remains hypothetically, with regard to the low potency.

The *Andrographis paniculata* (herba) tincture exhibited a weak antagonism (**Supplementary Table S2**). However, the tested labdane diterpenoid andrographolide, which possesses antiphlogistic, anticancer, and neuroprotective effects (Chan et al., 2010; Chun et al., 2010; Kou et al., 2014; Lin et al., 2014), is a partial antagonist of the GABA_A_ receptor (**Figure 5**; **Supplementary Table S4**). Moreover, the action of andrographolide appears to be non-competitive in nature (**Supplementary Figure S2**). The andrographolide content of the *A. paniculata* leaves was approximately 1% of the dry weight (Chao and Lin, 2010). Hence, the concentration in the applied tincture can be approximated (≈6 μM). Therefore, the antagonistic effect of the tincture is not solely due to the action of andrographolide.

The *Salvia miltiorrhiza* (radix) tincture exhibited the strongest antagonistic effect on the GABA_A_ receptor, exceeding a blocking effect of 80% (**Figure 3**; **Supplementary Table S2**). This compound is traditionally used for the treatment of cardiovascular diseases and ischemia, and it inhibits platelet aggregation via the action of tanshinones (Han et al., 2008). One specific tanshinone, miltirone, was identified as a ligand at the benzodiazepine binding site of the GABA_A_ receptor in the CNS, based on ligand binding assays (Lee et al., 1991). Nevertheless, miltirone failed to modulate the GABA-induced current of the GABA_A_ receptor in a recombinant expression system (*Xenopus* oocytes) as well as in cultured rat hippocampal pyramidal cells (Mostallino et al., 2004). Hence, the involvement of miltirone in the observed inhibition of GABA_A_ receptor responses by the *Salvia miltiorrhiza* tincture seems unlikely. Additionally, rosmarinic acid, a monoterpenoid from *Salvia miltiorrhiza* (Adams et al., 2006), only showed a slight inhibition of the GABA_A_ receptor in the screening (**Figure 4**; **Supplementary Table S3**) and therefore does not account for the strong antagonistic effect of the tincture.

*Salvia miltiorrhiza* and *Astragalus propinquus* are compounds in a two-ingredient intermixture called myelophil. This mixture has been shown to reduce chemotherapy-correlated adverse side effects, including myelosuppression and anemia, from the cytostatic drug fluorouracil (Shin et al., 2008) and possesses no described side effects itself (Jung et al., 2009). Furthermore, myelophil reduced fatigue in patients suffering from chronic fatigue (Cho et al., 2009) and exhibited anti-amnesic effects in scopolamine-treated mice (Lee et al., 2014). Even if the latter is presumably caused by the increased expression of ERKs and mAChR1 (Lee et al., 2014), a contribution of the inhibition of *Salvia miltiorrhiza* on the GABA_A_ receptor to the anti-fatigue effects of myelophil is likely, due to the increased neuronal excitability resulting from GABA_A_ receptor inhibition. In contrast to *Salvia miltiorrhiza*, *Astragalus propinquus* did not affect the GABA_A_ receptor in this study (**Supplementary Table S2**).

## Conclusion

We believe that a broad screening of the ethanol tinctures of herbal remedies via electrophysiological assays is a reliable method to obtain an overview of the pharmacological action of these compounds on clinically relevant receptors. In this study, a variety of tinctures were detected, whose pharmacological actions on the investigated receptors are in agreement with their traditional uses and physiological effects. For example, the antiemetic remedies *Zingiber officinalis* (rhizoma), *Panax ginseng* (radix), and *Syzygium aromaticum* (flos) were identified as antagonists of the 5-HT_3A_ receptor, and sedative drugs such as *Panax ginseng* (radix), *Scutellaria baicalensis* (radix), and *Mentha canadensis* (herba) were found as GABA_A_ receptor potentiating tinctures. Furthermore, *Lindera aggregata* (radix) and *Leonurus japonicus* (herba) are identified as potent 5-HT_3A_ receptor antagonists. Boldin was recently identified as a potent 5-HT_3A_ receptor blocker (Walstab et al., 2014) and is likely the active component of *Lindera aggregata* (radix). In this study, leonurine was identified as a new natural 5-HT_3A_ receptor antagonist. It inhibited the 5-HT_3A_ receptor with a similar potency to boldine. Furthermore, tinctures that antagonize the GABA_A_ receptor, were detected. E.g., *Salvia miltiorrhiza* (radix), which is part of a Kampo remedy called myelophil, blocks GABA_A_ receptor responses with high efficacy. Myelophil was previously shown to ameliorate fatigue symptoms. Therefore, we hypothesized that *Salvia miltiorrhiza* (radix) may contribute to the anti-fatigue effects of myelophil due to the increased neuronal excitability accompanied by the antagonism of the GABA_A_ receptor. Hence, electrophysiological screens are a helpful tool to understand the physiological effects of herbal drugs and enable the identification of new bioactive compounds, such as leonurine, for the development of new pharmaceutics or the more precise application of herbal remedies. The development of new pharmaceutical drugs out of the identified active ingredients of these herbs is a further possibility. The knowledge regarding the medicinal benefit of Kampo remedies has continued to evolve for centuries. This study can be thought of as an attempt to “translate” this traditional system into a contemporary, pharmacological system. Here, two classical drug targets were investigated, the 5-HT_3_ and GABA_A_ receptors. However, there is still little knowledge with regard to the pharmacological action of many Kampo remedies on other clinically relevant targets, and therefore, there is great potential for future research.

## Author Contributions

KH, HH, MW, and GG conceived and designed the experiments; KH, RH, PZ, and LB performed the experiments; PL contributed materials; KH, RH, and GG wrote the paper.

## Conflict of Interest Statement

The authors declare that the research was conducted in the absence of any commercial or financial relationships that could be construed as a potential conflict of interest.

## References

[B1] Adams J. D. Wang R. Yang J. Lien E. J. (2006). Preclinical and clinical examinations of *Salvia miltiorrhiza* and its tanshinones in ischemic conditions. *Chin. Med.* 1:3 10.1186/1749-8546-1-3 PMC1761145 17302964

[B2] Alexeev M. Grosenbaugh D. K. Mott D. D. Fisher J. L. (2012). The natural products magnolol and honokiol are positive allosteric modulators of both synaptic and extra-synaptic GABA(A) receptors. *Neuropharmacology* 62 2507–2514. 10.1016/j.neuropharm.2012.03.002 22445602 PMC3652012

[B3] Aoshima H. Hamamoto K. (1999). Potentiation of GABAA receptors expressed in *Xenopus* oocytes by perfume and phytoncid. *Biosci. Biotechnol. Biochem.* 63 743–748. 10.1271/bbb.63.743 10361687

[B4] Atawodi S. E. Atawodi J. C. Pfundstein B. Spiegelhalder B. Bartsch H. Owen R. (2011). Assessment of the polyphenol components and in vitro antioxidant properties of *Syzygium aromaticum* (L.) Merr. & Perry. *Electron.* *J. Environ. Agric. Food Chem.* 10 1970–1978.

[B5] Barann M. Linden I. Witten S. Urban B. W. (2008). Molecular actions of propofol on human 5-HT3A receptors: enhancement as well as inhibition by closely related phenol derivatives. *Anesth. Analg.* 106 846–857. 10.1213/ane.0b013e318162ca7c 18292429

[B6] Barann M. Meder W. Dorner Z. Bruss M. Bonisch H. Gothert M. (2000). Recombinant human 5-HT(3A) receptors in outside-out patches of HEK 293 cells: basic properties and barbiturate effects. *Naunyn Schmiedebergs Arch. Pharmacol.* 362 255–265. 10.1007/s002100000288 10997728

[B7] Bhowmik D. Kumar K. P. S. Yadav A. Srivastava S. Paswan S. Dutta A. S. (2012). Recent trends in Indian traditional herbs *Syzygium Aromaticum* and its health benefits. *J. Pharmacogn. Phytochem.* 1 6–17.

[B8] Calcaterra N. E. Barrow J. C. (2014). Classics in chemical neuroscience: Diazepam (Valium). *ACS Chem. Neurosci.* 5 253–260. 10.1021/cn5000056 24552479 PMC3990949

[B9] Cestari I. N. Uchida I. Li L. Burt D. Yang J. (1996). The agonistic action of pentobarbital on GABAA beta-subunit homomeric receptors. *Neuroreport* 7 943–947. 10.1097/00001756-199603220-00023 8724679

[B10] Chaieb K. Hajlaoui H. Zmantar T. Kahla-Nakbi A. B. Rouabhia M. Mahdouani K. (2007). The chemical composition and biological activity of clove essential oil, *Eugenia caryophyllata* (*Syzigium aromaticum* L. Myrtaceae): a short review. *Phytother. Res* 21 501–506. 10.1002/ptr.2124 17380552

[B11] Chan S. J. Wong W. S. F. Wong P. T. H. Bian J. S. (2010). Neuroprotective effects of andrographolide in a rat model of permanent cerebral ischaemia. *Br. J. Pharmacol.* 161 668–679. 10.1111/j.1476-5381.2010.00906.x 20880404 PMC2990163

[B12] Chao W.-W. Lin B.-F. (2010). Isolation and identification of bioactive compounds in *Andrographis paniculata* (Chuanxinlian). *Chin. Med.* 5:17 10.1186/1749-8546-5-17 PMC2881933 20465823

[B13] Chen C. X. Kwan C. Y. (2001). Endothelium-independent vasorelaxation by leonurine, a plant alkaloid purified from Chinese motherwort. *Life Sci.* 68 953–960. 10.1016/S0024-3205(00)00987-5 11213365

[B14] Cho J. H. Cho C. K. Shin J. W. Son J. Y. Kang W. Son C. G. (2009). Myelophil, an extract mix of Astragali Radix and Salviae Radix, ameliorates chronic fatigue: a randomised, double-blind, controlled pilot study. *Complement. Ther. Med.* 17 141–146. 10.1016/j.ctim.2008.11.003 19398067

[B15] Choi S. E. Choi S. Lee J. H. Whiting P. J. Lee S. M. Nah S. Y. (2003). Effects of ginsenosides on GABA(A) receptor channels expressed in *Xenopus* oocytes. *Arch. Pharm. Res.* 26 28–33. 10.1007/BF03179927 12568354

[B16] Chun J. Y. Tummala R. Nadiminty N. Lou W. Liu C. Yang J. (2010). Andrographolide, an herbal medicine, inhibits Interleukin-6 expression and suppresses prostate cancer cell growth. *Genes Cancer* 1 868–876. 10.1177/1947601910383416 21442031 PMC3063649

[B17] Connolly C. N. Wafford K. A. (2004). The Cys-loop superfamily of ligand-gated ion channels: the impact of receptor structure on function. *Biochem. Soc. Trans.* 32 529–534. 10.1042/BST0320529 15157178

[B18] Costedio M. M. Hyman N. Mawe G. M. (2007). Serotonin and its role in colonic function and in gastrointestinal disorders. *Dis. Colon Rectum* 50 376–388. 10.1007/s10350-006-0763-3 17195902

[B19] Cubeddu L. X. Pendergrass K. Ryan T. York M. Burton G. Meshad M. (1994). Efficacy of oral ondansetron, a selective antagonist of 5-HT3 receptors, in the treatment of nausea and vomiting associated with cyclophosphamide-based chemotherapies. Ondansetron Study Group. *Am. J. Clin. Oncol.* 17 137–146. 10.1097/00000421-199404000-00010 8141105

[B20] Das P. Dillon G. H. (2005). Molecular determinants of picrotoxin inhibition of 5-hydroxytryptamine type 3 receptors. *J. Pharmacol. Exp. Ther.* 314 320–328. 10.1124/jpet.104.080325.coexpression 15814570

[B21] Ding M. Leach M. J. Bradley H. (2013). A systematic review of the evidence for topical use of ginger. *Explore (NY).* 9 361–364. 10.1016/j.explore.2013.08.001 24199775

[B22] Doak G. J. Sawynok J. (1997). Formalin-induced nocicept1ve behavior and edema: involvement of multiple peripheral 5-hydroxytryptamine receptor subtypes. *Neuroscience* 80 939–949. 10.1016/S0306-4522(97)00066-3 9276504

[B23] Eberhard U. (2003). *Leitfaden Kampo-Medizin, Japanische Phytotherapie*, 1 Edn Munich: Elsevier GmbH.

[B24] Ernst E. Pittler M. H. (2000). Efficacy of ginger for nausea and vomiting: a systematic review of randomized clinical trials. *Br. J. Anaesth.* 84 367–371. 10.1093/oxfordjournals.bja.a013442 10793599

[B25] Gershon M. D. (2004). Review article: serotonin receptors and transporters – roles in normal and abnormal gastrointestinal motility. *Aliment. Pharmacol. Ther.* 20(Suppl. 7), 3–14. 10.1111/j.1365-2036.2004.02180.x 15521849

[B26] Guenette S. A. Beaudry F. Marier J. F. Vachon P. (2006). Pharmacokinetics and anesthetic activity of eugenol in male Sprague-Dawley rats. *J. Vet. Pharmacol. Ther.* 29 265–270. 10.1111/j.1365-2885.2006.00740.x 16846463

[B27] Gyermek L. (1995). 5-HT3 receptors: pharmacologic and therapeutic aspects. *J. Clin. Pharmacol.* 35 845–855. 10.1002/j.1552-4604.1995.tb04129.x 8786244

[B28] Hall A. C. Turcotte C. M. Betts B. A. Yeung W.Y. Agyeman A. S. Burk L. A. (2004). Modulation of human GABAA and glycine receptor currents by menthol and related monoterpenoids. *Eur. J. Pharmacol.* 506 9–16. 10.1016/j.ejphar.2004.10.026 15588619

[B29] Han Z. Zheng Y. Chen N. Luan L. Zhou C. Gan L. (2008). Simultaneous determination of four alkaloids in *Lindera aggregata* by ultra-high-pressure liquid chromatography-tandem mass spectrometry. *J. Chromatogr. A* 1212 76–81. 10.1016/j.chroma.2008.10.017 18951552

[B30] Haniadka R. Rajeev A. G. Palatty P. L. Arora R. Baliga M. S. (2012). (Ginger) as an anti-emetic in cancer chemotherapy: a review. *J. Altern. Complement. Med.* 18 440–444. 10.1089/acm.2010.0737 22540971

[B31] Hara K. Sata T. (2007). The effects of the local anesthetics lidocaine and procaine on glycine and gamma-aminobutyric acid receptors expressed in *Xenopus* oocytes. *Anesth. Analg.* 104 1434–1439. 10.1213/01.ane.0000261509.72234.a6 17513637

[B32] Herbrechter R. Ziemba P. M. Hoffmann K. M. Hanns H. Werner M. Gisselmann G. (2015). Identification of *Glycyrrhiza* as the rikkunshito constituent with the highest antagonistic potential on heterologously expressed 5-HT_3A_ receptors due to the action of flavonoids. *Front. Pharmacol.* 6:130 10.3389/fphar.2015.00130 PMC4490227 26191003

[B33] Hoffmann K. M. Beltran L. Ziemba P. M. Hatt H. Gisselmann G. (2016). Potentiating effect of glabridin from *Glycyrrhiza glabra* on GABAA receptors. *Biochem. Biophys. Rep.* 6 197–202. 10.1016/j.bbrep.2016.04.007 PMC5689168 29214227

[B34] Huang B. Yi B. Duan Y. Sun L. Yu X. Guo J. (2008). Characterization and expression profiling of tyrosine aminotransferase gene from *Salvia miltiorrhiza* (Dan-shen) in rosmarinic acid biosynthesis pathway. *Mol. Biol. Rep.* 35 601–612. 10.1007/s11033-007-9130-2 17805988

[B35] Huang Q. R. Iwamoto M. Aoki S. Tanaka N. Tajima K. Yamahara J. (1991). Anti-5-hydroxytryptamine3 effect of galanolactone, diterpenoid isolated from ginger. *Chem. Pharm. Bull. (Tokyo).* 39 397–399. 10.1248/cpb.39.397 2054863

[B36] Hui K. M. Huen M. S. Y. Wang H. Y. Zheng H. Sigel E. Baur R. (2002). Anxiolytic effect of wogonin, a benzodiazepine receptor ligand isolated from *Scutellaria baicalensis* Georgi. *Biochem. Pharmacol.* 64 1415–1424. 10.1016/S0006-2952(02)01347-3 12392823

[B37] Hunyadi A. Martins A. Hsieh T.-J. Seres A. Zupkó I. (2012). Chlorogenic acid and rutin play a major role in the in vivo anti-diabetic activity of *Morus alba* leaf extract on type II diabetic rats. *PLoS ONE* 7:e50619 10.1371/journal.pone.0050619 PMC3503931 23185641

[B38] Ip S. P. Mak D. H. Li P. C. Poon M. K. Ko K. M. (1996). Effect of a lignan-enriched extract of *Schisandra chinensis* on aflatoxin B1 and cadmium chloride-induced hepatotoxicity in rats. *Pharmacol. Toxicol.* 78 413–416. 10.1111/j.1600-0773.1996.tb00228.x 8829203

[B39] Jeggo R. D. Kellett D. O. Wang Y. Ramage A. G. Jordan D. (2005). The role of central 5-HT3 receptors in vagal reflex inputs to neurones in the nucleus tractus solitarius of anaesthetized rats. *J. Physiol.* 566 939–953. 10.1113/jphysiol.2005.085845 15905216 PMC1464782

[B40] Jung D. H. Choi E. J. Jeon H. H. Lee Y. H. Park H. (2014). Effects of GC7101 a novel prokinetic agent on gastric motor function: ex vivo study. *J. Neurogastroenterol. Motil.* 20 469–474. 10.5056/jnm14010 25273117 PMC4204422

[B41] Jung J.-M. Shin J.-W. Son J.-Y. Seong N.-W. Seo D.-S. Cho J.-H. (2009). Four-week repeated dose toxicity test for Myelophil in SD rats. *J. Korean Orient. Med.* 30 79–85.

[B42] Kessler A. Sahin-Nadeem H. Lummis S. C. R. Weigel I. Pischetsrieder M. Buettner A. (2014). GABAA receptor modulation by terpenoids from *Sideritis* extracts. *Mol. Nutr. Food Res.* 58 851–862. 10.1002/mnfr.201300420 24273211 PMC4384808

[B43] Kim J. H. Yoon I. S. Lee B. H. Choi S. H. Lee J. H. Lee J. H. (2005). Effects of Korean red ginseng extract on cisplatin-induced nausea and vomiting. *Arch. Pharm. Res.* 28 680–684. 10.1007/BF02969358 16042077

[B44] Kondo K. Shiba M. Nakamura R. Morota T. Shoyama Y. (2007). Constituent properties of licorices derived from *Glycyrrhiza uralensis*, *G. glabra*, or *G. inflata* identified by genetic information. *Biol. Pharm. Bull.* 30 1271–1277. 10.1248/bpb.30.1271 17603166

[B45] Kou W. Sun R. Wei P. Yao H.-B. Zhang C. Tang X.-Y. (2014). Andrographolide suppresses IL-6/Stat3 signaling in peripheral blood mononuclear cells from patients with chronic Rhinosinusitis with Nasal Polyps. *Inflammation* 37 1738–1743. 10.1007/s10753-014-9902-5 24803294

[B46] Lankiewicz S. Lobitz N. Wetzel C. H. Rupprecht R. Gisselmann G. Hatt H. (1998). Molecular cloning, functional expression, and pharmacological characterization of 5-hydroxytryptamine3 receptor cDNA and its splice variants from guinea pig. *Mol. Pharmacol.* 53 202–212. 9463477 10.1124/mol.53.2.202

[B47] Lee B. H. Kim H. J. Chung L. Nah S. Y. (2013). Ginsenoside Rg(3) regulates GABAA receptor channel activity: involvement of interaction with the gamma(2) subunit. *Eur. J. Pharmacol.* 705 119–125. 10.1016/j.ejphar.2013.02.040 23499684

[B48] Lee B. H. Lee J. H. Lee S. M. Jeong S. M. Yoon I. S. Lee J. H. (2007). Identification of ginsenoside interaction sites in 5-HT3A receptors. *Neuropharmacology* 52 1139–1150. 10.1016/j.neuropharm.2006.12.001 17257631

[B49] Lee B.-H. Pyo M. K. Lee J.-H. Choi S.-H. Shin T.-J. Lee S.-M. (2008). Differential regulations of quercetin and its glycosides on ligand-gated ion channels. *Biol. Pharm. Bull.* 31 611–617. 10.1248/bpb.31.611 18379051

[B50] Lee C. M. Wong H. N. Chui K. Y. Choang T. F. Hon P. M. Chang H. M. (1991). Miltirone, a central benzodiazepine receptor partial agonist from a Chinese medicinal herb *Salvia miltiorrhiza*. *Neurosci. Lett.* 127 237–241. 10.1016/0304-3940(91)90802-Z 1652718

[B51] Lee J. S. Kim H. G. Han J. M. Kim D. W. Yi M. H. Son S. W. (2014). Ethanol extract of Astragali Radix and Salviae Miltiorrhizae Radix, Myelophil, exerts anti-amnesic effect in a mouse model of scopolamine-induced memory deficits. *J. Ethnopharmacol.* 153 782–792. 10.1016/j.jep.2014.03.048 24690775

[B52] Li J. B. Ding Y. Li W. M. (2002). A new sesquiterpene from the roots of *Lindera strychnifolia*. *Chin. Chem. Lett.* 13 965–967. 10.1016/j.cclet.2009.07.014

[B53] Lin C.-C. Ng L. T. Hsu F.-F. Shieh D.-E. Chiang L.-C. (2004). Cytotoxic effects of *Coptis chinensis* and *Epimedium sagittatum* extracts and their major constituents (berberine, coptisine and icariin) on hepatoma and leukaemia cell growth. *Clin. Exp. Pharmacol. Physiol.* 31 65–69. 10.1111/j.1440-1681.2004.03951.x 14756686

[B54] Lin H.-C. Pan S.-M. Ding H.-Y. Chou T.-C. Chang W.-L. (2007). Antiplatelet Effect of Leonurine from *Leonurus sibiricus*. *Taiwan Pharm. J.* 69 149–152.

[B55] Lin H.-H. Shi M.-D. Tseng H.-C. Chen J.-H. (2014). Andrographolide sensitizes the cytotoxicity of human colorectal carcinoma cells toward cisplatin via enhancing apoptosis pathways in vitro and in vivo. *Toxicol. Sci.* 139 108–120. 10.1093/toxsci/kfu032 24563380

[B56] Lin M.-C. Hsu P.-C. Yin M.-C. (2013). Protective effects of *Houttuynia cordata* aqueous extract in mice consuming a high saturated fat diet. *Food Funct.* 4 322–327. 10.1039/c2fo30228d 23165792

[B57] Min K. T. Koo B. N. Kang J. W. Bai S. J. Ko S. R. Cho Z.-H. (2003). Effect of ginseng saponins on the recombinant serotonin type 3A receptor expressed in *Xenopus* oocytes: implication of possible application as an antiemetic. *J. Altern. Complement. Med.* 9 505–510. 10.1089/107555303322284794 14499026

[B58] Mostallino M. C. Mascia M. P. Pisu M. G. Busonero F. Talani G. Biggio G. (2004). Inhibition by miltirone of up-regulation of GABAA receptor α4 subunit mRNA by ethanol withdrawal in hippocampal neurons. *Eur. J. Pharmacol.* 494 83–90. 10.1016/j.ejphar.2004.04.021 15212961

[B59] Olsen R. W. Sapp D. M. Bureau M. H. Turner D. M. Kokka N. (1991). Allosteric actions of central nervous system depressants including anesthetics on subtypes of the inhibitory gamma-aminobutyric acidA receptor-chloride channel complex. *Ann. N. Y. Acad. Sci.* 625 145–154. 10.1111/j.1749-6632.1991.tb33838.x 1711804

[B60] Pan S.-Y. Dong H. Zhao X.-Y. Xiang C.-J. Fang H.-Y. Fong W.-F. (2008). Schisandrin B from Schisandra chinensis reduces hepatic lipid contents in hypercholesterolaemic mice. *J. Pharm. Pharmacol.* 60 399–403. 10.1211/jpp.60.3.0017 18284822

[B61] Panossian A. Wikman G. (2010). Effects of adaptogens on the central nervous system and the molecular mechanisms associated with their stress—protective activity. *Pharmaceuticals* 3 188–224. 10.3390/ph3010188 PMC3991026 27713248

[B62] Park H. G. Yoon S. Y. Choi J. Y. Lee G. S. Choi J. H. Shin C. Y. (2007). Anticonvulsant effect of wogonin isolated from *Scutellaria baicalensis*. *Eur. J. Pharmacol.* 574 112–119. 10.1016/j.ejphar.2007.07.011 17692312

[B63] Priestley C. M. Williamson E. M. Wafford K. A. Sattelle D. B. (2003). Thymol, a constituent of thyme essential oil, is a positive allosteric modulator of human GABA(A) receptors and a homo-oligomeric GABA receptor from *Drosophila melanogaster*. *Br. J. Pharmacol.* 140 1363–1372. 10.1038/sj.bjp.0705542 14623762 PMC1574153

[B64] Rauchensteiner F. Matsumura Y. Yamamoto Y. Yamaji S. Tani T. (2005). Analysis and comparison of Radix Glycyrrhizae (licorice) from Europe and China by capillary-zone electrophoresis (CZE). *J. Pharm. Biomed. Anal.* 38 594–600. 10.1016/j.jpba.2005.01.038 15967286

[B65] Saras A. Gisselmann G. Vogt-Eisele A. K. Erlkamp K. S. Kletke O. Pusch H. (2008). Histamine action on vertebrate GABAA receptors: direct channel gating and potentiation of GABA responses. *J. Biol. Chem.* 283 10470–10475. 10.1074/jbc.M709993200 18281286

[B66] Sergeeva O. A. Kletke O. Kragler A. Poppek A. Fleischer W. Schubring S. R. (2010). Fragrant dioxane derivatives identify β1-subunit-containing GABA a receptors. *J. Biol. Chem.* 285 23985–23993. 10.1074/jbc.M110.103309 20511229 PMC2911342

[B67] Shang X. Pan H. Wang X. He H. Li M. (2014). *Leonurus japonicus* Houtt.: ethnopharmacology, phytochemistry and pharmacology of an important traditional Chinese medicine. *J. Ethnopharmacol.* 152 14–32. 10.1016/j.jep.2013.12.052 24412548

[B68] Shao Q.-S. Zhang A. Ye W.-W. Guo H.-P. Hu R.-H. (2014). Fast determination of two atractylenolides in Rhizoma Atractylodis Macrocephalae by Fourier transform near-infrared spectroscopy with partial least squares. *Spectrochim. Acta A Mol. Biomol. Spectrosc.* 120 499–504. 10.1016/j.saa.2013.10.035 24211810

[B69] Sharma M. Rauniar G. Das B. (2012). Experimental study of various central nervous system effects of eugenol in mice and rats. *Health Renaiss.* 10 208–214. 10.1016/j.jneuroim.2010.05.031

[B70] Sherkheli M. A. Vogt-Eisele A. K. Bura D. Beltrán Márques L. R. Gisselmann G. Hatt H. (2010). Characterization of selective TRPM8 ligands and their structure activity response (S.A.R) relationship. *J. Pharm. Pharm. Sci.* 13 242–253. 10.18433/J3N88N 20816009

[B71] Shin J. W. Lee M. M. Son J. Y. Lee N. H. Cho C. K. Chung W. K. (2008). Myelophil, a mixture of Astragali Radix and Salviae Radix extract, moderates toxic side effects of fluorouracil in mice. *World J. Gastroenterol.* 14 2323–2328. 10.3748/wjg.14.2323 18416457 PMC2705085

[B72] Smith P. L. Maloney K. N. Pothen R. G. Clardy J. Clapham D. E. (2006). Bisandrographolide from *Andrographis paniculata* activates TRPV4 channels. *J. Biol. Chem.* 281 29897–29904. 10.1074/jbc.M605394200 16899456

[B73] Song Y. X. Liu S. P. Jin Z. Qin J. F. Jiang Z. Y. (2013). Qualitative and quantitative analysis of *Andrographis paniculata* by rapid resolution liquid chromatography/time-of-flight mass spectrometry. *Molecules* 18 12192–12207. 10.3390/molecules181012192 24084022 PMC6270035

[B74] Taferner B. Schuehly W. Huefner A. Baburin I. Wiesner K. Ecker G. F. (2011). Modulation of GABAA-receptors by honokiol and derivatives: subtype selectivity and structure-activity relationship. *J. Med. Chem.* 54 5349–5361. 10.1021/jm200186n 21699169

[B75] Takeda H. Sadakane C. Hattori T. Katsurada T. Ohkawara T. Nagai K. (2008). Rikkunshito, an herbal medicine, suppresses cisplatin-induced anorexia in rats via 5-HT2 receptor antagonism. *Gastroenterology* 134 2004–2013. 10.1053/j.gastro.2008.02.078 18439428

[B76] Tjong Y. Ip S. Lao L. Fong H. H. S. Sung J. J. Y. Berman B. (2011). Analgesic effect of *Coptis chinensis* rhizomes (Coptidis Rhizoma) extract on rat model of irritable bowel syndrome. *J. Ethnopharmacol.* 135 754–761. 10.1016/j.jep.2011.04.007 21511022 PMC3100428

[B77] Tominaga K. Kido T. Ochi M. Sadakane C. Mase A. Okazaki H. (2011). The traditional japanese medicine rikkunshito promotes gastric emptying via the antagonistic action of the 5-HT 3 receptor pathway in rats. *Evid. Based Complement. Altern. Med.* 2011:248481 10.1093/ecam/nep173 PMC3095508 19861508

[B78] Ueta K. Suzuki T. Sugimoto M. Uchida I. Mashimo T. (2007). Local anesthetics have different mechanisms and sites of action at recombinant 5-HT3 receptors. *Reg. Anesth. Pain Med.* 32 462–470. 10.1016/j.rapm.2007.06.391 18035290

[B79] Walstab J. Krüger D. Stark T. Hofmann T. Demir I. E. Ceyhan G. O. (2013). Ginger and its pungent constituents non-competitively inhibit activation of human recombinant and native 5-HT3 receptors of enteric neurons. *Neurogastroenterol. Motil.* 25 439–447,e302. 10.1111/nmo.12107 23490018

[B80] Walstab J. Wohlfarth C. Hovius R. Schmitteckert S. Röth R. Lasitschka F. (2014). Natural compounds boldine and menthol are antagonists of human 5-HT3 receptors: implications for treating gastrointestinal disorders. *Neurogastroenterol. Motil.* 26 810–820. 10.1111/nmo.12334 24708203

[B81] Wang F. Jiang Y.-P. Wang X.-L. Lin S. Pu P.-B. Zhu C.-G. (2013). Chemical constituents from flower buds of *Lonicera japonica*. *China J. Chin. Mater. Med.* 38 1378–1385. 10.4268/cjcmm20130924 23944073

[B82] Wang J. Zhao Y.-M. Zhang M.-L. Shi Q. (2014). Simultaneous determination of chlorogenic acid, caffeic acid, alantolactone and isoalantolactone in *Inula helenium* by HPLC. *J. Chromatogr. Sci.* 53 526–530. 10.1093/chromsci/bmu079 24996657

[B83] Wang Z. Zhang P.-L. Ju Y. (2004). Effect of leonurine on the activity of creatine kinase. *J. Asian Nat. Prod. Res.* 6 281–287. 10.1080/10286020310001595962 15621588

[B84] Watanabe K. Matsuura K. Gao P. Hottenbacher L. Tokunaga H. Nishimura K. (2011). Traditional japanese kampo medicine: clinical research between modernity and traditional medicine-the state of research and methodological suggestions for the future. *Evid. Based Complement. Altern. Med.* 2011:513842 10.1093/ecam/neq067 PMC3114407 21687585

[B85] Wei B. Li Q. Fan R. Su D. Chen X. Jia Y. (2013). Determination of monoamine and amino acid neurotransmitters and their metabolites in rat brain samples by UFLC-MS/MS for the study of the sedative-hypnotic effects observed during treatment with *S. chinensis*. *J. Pharm. Biomed. Anal.* 10 416–422. 10.1016/j.jpba.2013.09.022 24176746

[B86] World Health Organization (2005). *WHO Monographs on Selected Medicinal Plants. World Health 4.* Available at: http://kamillaviragzat.hu/wtDocument/browse/root/szakmai-anyagok/WHO_Monographs_vol1.pdf

[B87] Xiang Y.-Z. Shang H.-C. Gao X.-M. Zhang B.-L. (2008). A comparison of the ancient use of Ginseng in traditional Chinese medicine with moder pharmacological experiments and clinical trials. *Phytother. Res.* 22 851–858. 10.1002/ptr.2384 18567057

[B88] Yadav A. V. Kawale L. A. Nade V. S. (2008). Effect of *Morus alba* L. (mulberry) leaves on anxiety in mice. *Indian J. Pharmacol.* 40 32–36. 10.4103/0253-7613.40487 21264159 PMC3023120

[B89] Zhang C. Mao X. Zhao X. Liu Z. Liu B. Li H. (2014). Gomisin N isolated from *Schisandra chinensis* augments pentobarbital-induced sleep behaviors through the modification of the serotonergic and GABAergic system. *Fitoterapia* 96 123–130. 10.1016/j.fitote.2014.04.017 24785966

[B90] Zhao B. Kim E. J. Son K. H. Son J. K. Min B. S. Woo M. H. (2015). Quality evaluation and pattern recognition analyses of marker compounds from five medicinal drugs of Rutaceae family by HPLC/PDA. *Arch. Pharm. Res.* 38 1512–1520. 10.1007/s12272-015-0583-x 25732613

[B91] Ziemba P. M. Schreiner B. S. P. Flegel C. Herbrechter R. Stark T. D. Hofmann T. (2015). Activation and modulation of recombinantly expressed serotonin receptor type 3A by terpenes and pungent substances. *Biochem. Biophys. Res. Commun.* 467 1090–1096. 10.1016/j.bbrc.2015.09.074 26456648

