# Supplementary material for: Kampo Medicine: Evaluation of the Pharmacological Activity of 121 Herbal Drugs on GABAA and 5-HT3A Receptors
Source: Front Pharmacol. 2016 Jul 29;7:219. doi: 10.3389/fphar.2016.00219 (PMC4965468; doi:10.3389/fphar.2016.00219)
Supplement: Supplementary file 1 [file Data_Sheet_1.PDF]

Supplementary Material

Kampo medicine: Evaluation of the pharmacological activity of 121 herbal drugs on $GABA_A$ and 5-HT_{3A} receptors

Katrin M. Hoffmann[1], Robin Herbrechter[1], Paul M. Ziemba[1], Peter Lepke[2], Leopoldo Beltrán[1], Hanns Hatt[1], Markus Werner[1,3] and Günter Gisselmann[1]

Affiliation

[1]Department of Cell Physiology, Ruhr-University Bochum, Bochum, Germany

[2]Kronen Apotheke Wuppertal, Wuppertal, Germany

[3]present address: Department of Biochemistry I - Receptor Biochemistry, Ruhr University Bochum Bochum, Germany

Correspondence:

Günter Gisselmann

Ruhr-University Bochum

Department of Cell Physiology

ND-4/165; Universitätsstraße 150

D-44780 Bochum, Germany

E-mail address: guenter.gisselmann@rub.de

1. Supplementary Tables

Supplementary Table 1. Examined Kampo remedies

Botanical name:	Part of plant:	Japanese name:	Chinese name:	Synonyme:
Achyranthes bidentata Blume, *Amaranthaceae*	radix	gostsu	Huai Niu Xi	*Achyranthis bid. rad.*
Aconitum coreanum (H.Lév.) Rapaics, *Ranunculaceae*	radix	bushi	Fu Zi	*Aconit. carm. lat. praep. rad.*
Actaea cimicifuga L., *Ranunculaceae*	rhizoma	shoma	Sheng Ma	*Cimicifuga her. rhiz.*
Alisma plantago-aquatica subsp. orientale (Sam.) Sam., *Alismataceae*	rhizoma	takusha	Ze Xie	*Alismatis or. rhiz.*
Alpinia officinarum Hance, *Zingiberaceae*	rhizoma	ryokyo	Gao Liang Jiang	*Alpinia off. rhiz.*
Amomum villosum Lour., *Zingiberaceae*	fructus	shukusha	Sha Ren	*Amomum vill. fruct.*
Andrographis paniculata (Burm.f.) Nees, *Acanthaceae*	herba		Chuan Xin Lian	*Andrographitis herb.*
Anemarrhena asphodeloides Bunge, *Asparagaceae*	rhizoma	chimo	Zhi Mu	*Anemarrhena asp. rhiz.*
Angelica dahurica (Hoffm.) Benth. & Hook.f. ex Franch. & Sav., *Apiaceae*	radix	byakushi	Bai Zhi	*Angelica dah. rad.*
Angelica pubescens Maxim., Apiaceae	radix	dokkatsu	Du Huo	*Angelica pub. rad. = Heraclei rad.*
Angelica sinensis (Oliv.) Diels, *Apiaceae*	radix	toki	Dang Gui	*Angelica sin. rad*
Arctium lappa L., *Compositae*	fructus	goboshi	Niu Bang Zi	*Arctium lappa fruct.*
Areca catechu L., *Arecaceae*	semen	binroshi	Bing Lang	*Areca cat. sem.*
Areca catechu L., *Arecaceae*	pericarpium	daikukuhi	Da Fu Pi	*Areca cat. pericarpium*
Arisaema consanguineum Schott, *Araceae*	rhizoma	tennansho	Tian Nan Xing	*Arisaematis erub. rhiz.*
Armeniaca amarella Schur, *Rosaceae*	semen	kyonin	Ku Xing Ren	*Armeniacae am. sem.*
Artemisia argyi H.Lév. & Vaniot, *Compositae*	herba	gaiyo	Ai Ye	*Artemisia argyi herb.*
Asparagus cochinchinensis (Lour.) Merr., *Asparagaceae*	radix	tenmondo	Tian Dong	*Asparagus coch. rad.*
Astragalus propinquus Schischkin., *Leguminosae*	radix	ogi	Huang Qi	*Astragali mem. rad.*
Atractylodes lancea (Thunb.) DC., *Compositae*	rhizoma	sojutsu	Cang Zhu	*Atractylodis lanc. rhiz.*
Atractylodes macrocephala Koidz., *Compositae*	rhizoma	byakujutsu	Bai Zhu	*Atractylodis mac. rhiz*

Aucklandia lappa DC., *Compositae*	radix		Mu Xiang	*Aucklandia lap. rad.*
Bambusa tuldoides Munro; *Phyllostachys nigra* (Lodd. ex Lindl.) Munro; u.a., *Poaceae*	caulis	chikujo	Zhu Ru	*Bambusa caul. in taeniam*
Boswellia sacra Flueck., *Burseraceae*	resinae		Ru Xiang	*Olibanum*
Bupleurum chinense DC., *Apiaceae*	radix	saiko	Chai Hu	*Bupleurum chin. rad.*
Caesalpinia sappan L., *Leguminosae*	lignum		Su Mu	*Sappalum lign.*
Cannabis sativa L., *Cannabaceae*	semen	soboku	Huo Ma Ren	*Cannabis sat. sem.*
Carthamus tinctorius L., *Compositae*	flos	koka	Hong Hua	*Carthamus tinct. flos*
Chaenomeles speciosa (Sweet) Nakai, *Rosaceae*	fructus	mokka	Mu Gua	*Chaenomelis spect. fruct.*
Chrysanthemum morifolium Ramat., *Compositae*	flos	kikuka	Ju Hua	*Chrysanthemi morifol. flos*
Cinnamomum cassia (L.) J.Presl, *Lauraceae*	cortex	keihi	Rou Gui	*Cinnamomum cas. cort.*
Cinnamomum cassia (L.) J.Presl, *Lauraceae*	ramus	keishi	Gui Zhi Cassia	*Cinnamomum cassia ram.*
Cistanche deserticola Y.C.Ma, *Orobanchaceae*	herba	nikujuyo	Rou Cong Rong	*Cistanches caulis = Cistanches herb.*
Citrus × aurantium L., *Rutaceae*	fructus	kikoku	Zhi Qiao/ Zhi Ke	*Aurantii fruct. = Citri aurant*
Citrus reticulata Blanco, *Rutaceae*	pericarpium	chinpi	Chen Pi	*Aurantii pericarp.*
Citrus reticulata Blanco, *Rutaceae*	Pericarpium, vir.	seihi	Qing Pi	*Aurantii imm. per. = Citri ret. vir. per.*
Citrus trifoliata L.; *Citrus × aurantium* L.; u.a., *Rutaceae*	fructus	kijitsu	Zhi Shi	*Aurantii imm. fruct.*
Clematis armandii Franch., *Ranunculaceae*	caulis		Chuan Mu Tong	*Clematis arm. caul.=Mutong caulis=Horqua.*
Clematis chinensis Osbeck, *Ranunculaceae*	radix	ireisan	Wei Ling Xian	*Clematis chin. rad.*
Cnidium monnieri (L.) Cusson, *Apiaceae*	fructus	jashoshi	She Chuang Zi	*Cnidium mon. fruct.*
Coix lacryma-jobi L., *Poaceae*	semen	yokuinin	Yi Yi Ren	*Coix lac. sem.*
Combretum indicum (L.) DeFilipps, *Combretacea*	fructus	shikunshi	Shi Jun Zi	*Quisqualis fruct.*
Coptis chinensis Franch., *Ranunculaceae*	radix	oren	Huang Lian	*Coptis chin. rad.*
Cornus officinalis Siebold & Zucc., *Cornaceae*	fructus	sanshuyu	Shan Zhu Yu	*Cornus off. fruct.*
Corydalis yanhusuo (Y.H.Chou & Chun C.Hsu) W.T.Wang ex Z.Y.Su & C.Y.Wu, *Papaveraceae*	rhizoma	engosaku	Yan Hu Suo	*Corydalis turt. rhiz.*
Crataegus pinnatifida Bunge, *Rosaceae*	fructus	sanzashi	Shan Zha	*Crataegus pinnat. fruct.*
Curcuma aromatica Salisb., *Zingiberaceae*	radix	ukon	Yu Jin	*Curcuma wen. rad.*
Cyperus rotundus L., *Cyperaceae*	rhizoma	kobushi	Xiang Fu	*Cyperus rot. rhiz.*

Dimocarpus longan Lour., *Sapindaceae*	arillus	ryuganniku	Long Yan Rou	*Longan arillus*
Dioscorea oppositifolia L., *Dioscoreaceae*	rhizoma	sanyaku	Shan Yao	*Batatitis rhiz.*
Eleutherococcus nodiflorus (Dunn) S.Y.Hu, *Araliaceae*	cortex	gokahi	Wu Jia Pi	*Acanthopanax grac. cort*
Ephedra sinica Stapf, *Ephedraceae*	herba	mao	Ma Huang	*Ephedra sin. herb.*
Epimedium brevicornu Maxim., *Berberidaceae*	herba	inyokaku	Yin Yang Huo	*Epimedium brev. herb.*
Eriobotrya japonica (Thunb.) Lindl., *Rosaceae*	folia	biwayo	Pa Ye	*Eriobotrya jap. fol.*
Eucommia ulmoides Oliv., *Eucommiaceae*	cortex	tochû	Du Zhong	*Eucommia ulm cort.*
Foeniculum vulgare Mill., *Apiaceae*	fructus	uikyo	Xiao Hui Xiang	*Foeniculi fruct.*
Forsythia suspensa (Thunb.) Vahl, *Oleaceae*	fructus	rengyo	Lian Qiao	*Forsythia sus. fruct.*
Fritillaria verticillata Willd., *Liliaceae*	bulbus	(setsu) baimo	Zhe Bei Mu	*Fritillaria thun. bulb. Z*
Gardenia jasminoides J.Ellis, *Rubiaceae*	fructus	sanshishi	Zhi Zi	*Gardenia jasmin fruct.*
Gastrodia elata Blume, *Orchidaceae*	rhizoma	tenma	Tian Ma	*Gastrodia elata rhiz.*
Gentiana macrophylla Pall., *Gentianaceae*	radix	jingyo	Qin Jiao	*Gentiana macroph. rad.*
Gentiana manshurica Kitag., *Gentianaceae*	radix	ryûtan	Long Dan	*Gentiana scab. rad. = Gentiana mans. rad.*
Glehnia littoralis F.Schmidt ex Miq., *Apiaceae*	radix	hamabofu	Bei Sha Shen	*Glehnia litt. rad.*
Glycyrrhiza uralensis Fisch., *Leguminosae*	radix	kanzo	Gan Cao	*Glycyrrhiza ural. rad.*
Houttuynia cordata Thunb., *Saururaceae*	herba	jujaku	Yu Xing Cao	*Houttuynia cord. herb.*
Cryptotympana atrata (Fabricius, 1775)*	periostracum	sentai	Chan Tui	*Cicadae periost.*
Inula helenium L., *Compositae*	radix		Tu Mu Xiang	*Inula hel. rad.*
Inula japonica Thunb., *Compositae*	flos	senpukuka	Xuan Fu Hua	*Inula jap. Flos*
Leonurus japonicus Houtt., *Lamiaceae*	herba	yakumoso	Yi Mu Cao	*Leonurus heterophyllus herb.*
Ligusticum striatum DC., *Apiaceae*	rhizoma	senkyu	Chuan Xiong	*Ligusticum chuan. rhiz. = Chuanxiong rhiz.*
Lilium brownii F.E.Br. ex Miellez, *Liliaceae*	bulbus	byakugo	Bai He	*Lilium lanc. bulb.*
Lindera aggregata (Sims) Kosterm., *Lauraceae*	radix	uyaku	Wu Yao	*Lindera strych. rad.*
Lonicera japonica Thunb., *Caprifoliaceae*	caulis	nindo	Ren Dong Teng	*Lonicera jap. caulis*
Lonicera japonica Thunb., *Caprifoliaceae*	flos	kinginka	Jin Yin Hua	*Lonicera jap. flos.*
Lycium barbarum L., *Solanaceae*	fructus	kukoshi	Gou Qui Zi	*Lycium barb. fruct.*

Lycium chinense Mill.; *Lycium barbarum* L.; u.a., *Solanaceae*	cortex	jikoppi	Di Gu Pi	*Lycium barb. cort.*
Magnolia biondii Pamp., *Magnoliaceae*	flos	shini	Xin Yi	*Magnolia biond. flos.*
Magnolia officinalis Rehder & E.H.Wilson, *Magnoliaceae*	cortex	koboku	Hou Po	*Magnolia off. cort.*
Mentha canadensis L., *Lamiaceae*	herba	hakka	Bo He	*Mentha hap. herb.*
Morus alba L., *Moraceae*	cortex	sohakuhi	Sang Bai Pi	*Morus alba cort.*
Morus alba L., *Moraceae*	folia	soyo	Sang Ye	*Morus alba fol.*
Nelumbo nucifera Gaertn., *Nelumbonaceae*	semen	renniku	Lian Zi	*Nelumbo nuc. sem.*
Notopterygium incisum K.C.Ting ex H.T.Chang, *Apiaceae*	rhizoma	kyokatsu	Qiang Huo	*Notopterygium inc. rhiz.*
Ophiopogon japonicus (Thunb.) Ker Gawl., *Asparagaceae*	radix	bakumondo	Mai Dong	*Ophiopogon jap. rad.*
Paeonia × suffruticosa Andrews, *Paeoniaceae*	cortex	botanpi	Mu Dan Pi	*Moutan cortex*
Paeonia lactiflora Pall., *Paeoniaceae*	radix	shakuyaku	Bai Shao	*Paeonia alba = lactiflora rad.*
Panax ginseng C.A.Mey., *Araliaceae*	rhizoma	ninjin	Ren Shen	*Ginseng white*
Panax ginseng C.A.Mey., *Araliaceae*	rhizoma		Hong Shen	*Ginseng red. = Panax Ginseng*
Perilla frutescens (L.) Britton, *Lamiaceae*	folia	shisoyo	Zi Su Ye	*Perilla frut. fol.*
Peucedanum praeruptorum Dunn, *Apiaceae*	radix	kyokatsu	Qian Hu	*Peucedanum praer. rad.*
Phellodendron chinense C.K.Schneid., *Rutaceae*	cortex	obaku	Huang Bai	*Phellodendron chinense cort.*
Pinellia ternata (Thunb.) Makino, *Araceae*	rhizoma	hange	Ban Xia	*Pinellia tern. rhiz.*
Plantago asiatica L., *Plantaginaceae*	semen	shazenshi	Che Qian Zi	*Plantago asiatica sem.*
Platycladus orientalis (L.) Franco, *Cupressaceae*	folia	sokuhakuyo	Ce Bai Ye	*Biota Curcumen*
Platycodon grandiflorus (Jacq.) A.DC., *Campanulaceae*	radix	kikyo	Jie Geng	*Platycodus grand. rad.*
Pogostemon cablin (Blanco) Benth., *Lamiaceae*	herba		Guang Huo Xiang	*Pogostemon cabl. herb.* = Patchouli
Polygala tenuifolia Willd., *Polygalaceae*	radix	onji	Yuan Zhi	*Polygala ten. rad.*
Polygonatum kingianum Collett & Hemsl., *Asparagaceae*	rhizoma	osei	Huang Jing	*Polygonatum king. rhiz.*
Polyporus umbellatus (Pers.) Fires, *Polyporaceae*	sclerotium	chorei	Zhu Ling	*Polyporus umb.*
Prunella vulgaris L., *Lamiaceae*	spica	kagoso	Xia Ku Cao	*Prunella vulg. spica*
Prunus persica (L.) Batsch, *Rosaceae*	semen	tonin	Tao Ren	*Persica sem. = Prunus pers. sem.*
Pueraria montana (Lour.) Merr., *Leguminosae*	radix	kakkon	Ge Gen	*Pueraria lob. rad.*

Rehmannia glutinosa (Gaertn.) DC., *Plantaginaceae*	radix	jio	Di Huang/ Sheng Di Huang	*Rehmania glut. vir. rad.*
Reynoutria multiflora (Thunb.) Moldenke, *Polygonaceae*	radix	kashu	He Shou Wu	*Polygonum mult. rad.*
Rheum palmatum L., *Polygonaceae*	rhizoma	daio	Da Huang	*Rheum palm. rhiz.*
Salvia miltiorrhiza Bunge, *Lamiaceae*	radix	Tan	Dan Shen	*Salvia mil. rad.*
Saposhnikovia divaricata (Turcz.) Schischk., *Apiaceae*	radix	bofu	Fang Feng	*Saposhnikovia. rad.*
Schisandra chinensis (Turcz.) Baill., *Schisandraceae*	fructus	gomishi	Wu Wei Zi	*Schisandra chin. fruct.*
Scutellaria baicalensis Georgi, *Lamiaceae*	radix	ogon	Huang Qin	*Scutellaria baic. rad.*
Senna tora (L.) Roxb., *Leguminosae*	semen	ketsumeishi	Jue Ming Zi	*Cassia obt. sem. = Cassia toria sem.*
Syzygium aromaticum (L.) Merr. & L.M.Perry, *Myrtaceae*	flos	choji	Ding Xiang	*Caryopylli flos*
Terminalia chebula Retz., *Combretaceae*	fructus	kashi	He Zi	*Chebula fruct.*
Tetradium ruticarpum (A.Juss.) T.G.Hartley, *Rutaceae*	fructus	goshuyu	Wu Zhu Yu	*Evodia rut. fruct.*
Tussilago farfara L., *Compositae*	flos	kantoka	Kuan Dong Hua	*Farfarae flos*
Uncaria rhynchophylla (Miq.) Miq. ex Havil., *Rubiaceae*	ramus cum uncis	chotoko	Gou Teng	*Uncaria rhym. ram. cum uncis*
Wolfiporia extensa (Peck) Ginns, *Polyporaceae*	fructus corpore	bukuryo	Fu Ling	*Poria cocos*
Zanthoxylum bungeanum Maxim., *Rutaceae*	fructus	sansho	Chuan Jiao	*Zanthoxyli fruct.*
Zingiber officinale Roscoe, *Zingiberaceae*	rhizoma, sicc.	kankyo	Gan Jiang	*Zingiber off. sicc.*
Zingiber officinale Roscoe, *Zingiberaceae*	rhizoma, vir.	shokyo	Sheng Jiang	*Zingiber off. vir.*
Ziziphus jujuba Mill., *Rhamnaceae*	fructus	taiso	Da Zao	*Jujubae fruct.*
Ziziphus spina-christi (L.) Desf., *Rhamnaceae*	semen	sansonin	Suan Zao Ren	*Ziziphus spin. sem.*

* The only non-herbal drug (Arthropoda, Insecta, Cicadidae)

Supplementary Table 2. Action of Kampo tinctures on the 5-HT_{3A} and $GABA_A$ receptors

kampo remedy:	direct activation (0.1 Vol.-%) non-injected oocytes (n = 3)			direct activation (0.1 Vol.-%) injected oocytes (n = 3-8)						modulation (0.1 Vol.-%) injected oocytes (n = 3-8)					
	I [µA]			$I/I_{GABA\ (100\ \mu M)}$			$I/I_{5\text{-}HT\ (5\ \mu M)}$			$I/I_{GABA\ (10\ \mu M)}$			$I/I_{5\text{-}HT\ (5\ \mu M)}$		
	**Mean**	**±**	**SEM**	**Mean**	**±**	**SEM**	**Mean**	**±**	**SEM**	**Mean**	**±**	**SEM**	**Mean**	**±**	**SEM**
Achyranthes bidentata (radix)	-0.0040	±	0.0040	0.1291	±	0.0478	0.0058	±	0.0028	0.823	±	0.033	0.975	±	0.023
Aconitum coreanum (radix)	0.0090	±	0.0049	0.0224	±	0.0033	0.1199	±	0.0467	1.077	±	0.055	0.857	±	0.053
Actaea cimicifuga (rhizoma)	0.0000	±	0.0000	0.0085	±	0.0001	0.0018	±	0.0008	1.039	±	0.067	1.066	±	0.104
Alisma plantago-aquatica (rhizoma)	0.0000	±	0.0000	0.0205	±	0.0008	0.0024	±	0.0007	0.964	±	0.042	0.977	±	0.132
Alpinia officinarum (rhizoma)	-0.0017	±	0.0018	0.0175	±	0.0100	0.0039	±	0.0023	0.871	±	0.025	0.840	±	0.058
Amomum villosum (fructus)	0.0000	±	0.0010	0.0120	±	0.0098	0.0216	±	0.0157	0.829	±	0.061	0.831	±	0.035
Andrographis paniculata (herba)	-0.0027	±	0.0018	0.0168	±	0.0080	0.0040	±	0.0018	0.832	±	0.028	1.012	±	0.070
Anemarrhena asphodeloides (rhizoma)	0.0013	±	0.0024	0.0231	±	0.0103	0.0125	±	0.0082	0.836	±	0.054	0.808	±	0.103
Angelica dahurica (radix)	0.0000	±	0.0006	0.0392	±	0.0287	0.0020	±	0.0007	1.065	±	0.047	0.978	±	0.021
Angelica pubescens (radix)	-0.0017	±	0.0007	0.0157	±	0.0076	0.0682	±	0.0500	0.724	±	0.050	0.998	±	0.075
Angelica sinensis (radix)	-0.0023	±	0.0017	0.0668	±	0.0519	0.0869	±	0.0253	0.805	±	0.015	0.963	±	0.037
Arctium lappa (fructus)	0.0030	±	0.0010	0.0017	±	0.0013	0.0046	±	0.0012	0.957	±	0.062	0.850	±	0.018
Areca catechu (pericarpium)	-0.0027	±	0.0012	0.0181	±	0.0146	0.0046	±	0.0032	0.717	±	0.052	1.064	±	0.075
Areca catechu (semen)	0.0057	±	0.0032	0.0019	±	0.0001	0.0044	±	0.0022	0.878	±	0.091	1.072	±	0.070
Arisaema consanguineum (rhizoma)	0.0043	±	0.0022	0.0007	±	0.0048	0.0055	±	0.0011	0.885	±	0.111	0.987	±	0.013
Armeniaca amarella (semen)	-0.0017	±	0.0003	0.0982	±	0.0288	0.0029	±	0.0006	0.942	±	0.057	1.025	±	0.033
Artemisia argyi (herba)	0.0000	±	0.0000	0.0112	±	0.0025	0.0023	±	0.0005	1.063	±	0.130	0.852	±	0.310
Asparagus cochinchinensis (radix)	0.0033	±	0.0022	0.0091	±	0.0063	0.0021	±	0.0009	1.000	±	0.086	0.913	±	0.137
Astragalus propinquus (radix)	0.0043	±	0.0031	0.0133	±	0.0006	0.0047	±	0.0018	0.955	±	0.074	1.339	±	0.121
Atractylodes lancea (rhizoma)	-0.0003	±	0.0015	0.0141	±	0.0104	0.0021	±	0.0009	0.721	±	0.028	0.814	±	0.030
Atractylodes macrocephala (rhizoma)	0.0007	±	0.0020	0.0282	±	0.0230	0.0050	±	0.0016	0.871	±	0.014	0.903	±	0.061
Aucklandia lappa (radix)	0.0018	±	0.0001	0.0031	±	0.0008	0.0069	±	0.0045	0.972	±	0.122	0.772	±	0.180
Bambusa tuldoides (caulis)	0.0033	±	0.0026	0.0007	±	0.0001	0.0039	±	0.0028	1.093	±	0.078	1.272	±	0.148
Boswellia sacra	-0.0023	±	0.0009	0.0089	±	0.0036	0.0437	±	0.0355	0.941	±	0.044	1.097	±	0.181
Bupleurum chinense (radix)	-0.0007	±	0.0009	0.0230	±	0.0174	0.0024	±	0.0016	0.808	±	0.071	0.878	±	0.040
Cannabis sativa (semen)	0.0010	±	0.0033	0.0070	±	0.0054	0.0020	±	0.0012	1.051	±	0.032	1.241	±	0.130
Carthamus tinctorius (flos)	0.0000	±	0.0000	0.0124	±	0.0011	0.0065	±	0.0035	1.085	±	0.037	1.300	±	0.205

Chaenomeles speciosa (fructus)	0.0020	±	0.0017	0.0064	±	0.0163	0.0004	±	0.0003	0.978	±	0.014	1.373	±	0.197
Chrysanthemum morifolium (flos)	0.0017	±	0.0007	0.0048	±	0.0024	0.0012	±	0.0005	0.842	±	0.050	0.973	±	0.131
Cinnamomum cassia (cortex)	-0.0003	±	0.0007	0.0220	±	0.0197	0.0037	±	0.0015	0.777	±	0.048	0.852	±	0.068
Cinnamomum cassia (ramus)	0.0010	±	0.0016	0.0005	±	0.0006	0.0005	±	0.0003	1.061	±	0.089	1.123	±	0.118
Cistanche deserticola (herba)	0.0017	±	0.0012	0.0006	±	0.0038	0.0015	±	0.0007	0.948	±	0.054	1.082	±	0.111
Citrus × aurantium (fructus)	-0.0043	±	0.0003	0.0379	±	0.0253	0.0008	±	0.0003	0.725	±	0.044	0.958	±	0.077
Citrus reticulata (pericarpium)	0.0000	±	0.0000	0.0020	±	0.0013	0.0101	±	0.0059	0.883	±	0.070	0.883	±	0.066
Citrus reticulata (pericarpium, vir.)	0.0050	±	0.0037	0.0012	±	0.0004	0.0057	±	0.0026	0.966	±	0.076	0.979	±	0.148
Citrus trifoliata (fructus)	0.0143	±	0.0043	0.0424	±	0.0027	0.0018	±	0.0004	1.109	±	0.058	1.115	±	0.062
Clematis armandii (caulis)	-0.0010	±	0.0010	0.0033	±	0.0014	0.0012	±	0.0008	1.578	±	0.305	1.151	±	0.207
Clematis chinensis (radix)	0.0017	±	0.0015	0.0078	±	0.0005	0.0026	±	0.0019	0.959	±	0.042	1.061	±	0.260
Cnidium monnieri (fructus)	0.0010	±	0.0013	0.0013	±	0.0003	0.0009	±	0.0004	1.090	±	0.065	1.239	±	0.105
Coix lacryma-jobi (semen)	0.0037	±	0.0090	0.0005	±	0.0029	0.0074	±	0.0057	0.998	±	0.135	1.119	±	0.213
Combretum indicum (fructus)	-0.0020	±	0.0017	0.0416	±	0.0328	0.0016	±	0.0006	0.759	±	0.036	0.935	±	0.048
Coptis chinensis (radix)	0.0027	±	0.0002	0.0088	±	0.0008	0.0020	±	0.0009	0.903	±	0.068	0.349	±	0.171
Cornus officinalis (fructus)	-0.0007	±	0.0003	0.0128	±	0.0067	0.0026	±	0.0010	0.827	±	0.049	1.098	±	0.327
Corydalis yanhusuo (rhizoma)	0.0000	±	0.0000	0.0025	±	0.0020	0.0086	±	0.0055	1.120	±	0.052	0.955	±	0.067
Crataegus pinnatifida (fructus)	-0.0007	±	0.0009	0.0016	±	0.0007	0.0078	±	0.0061	0.845	±	0.045	1.142	±	0.314
Curcuma aromatica (radix)	-0.0020	±	0.0012	0.0027	±	0.0023	0.0012	±	0.0006	0.844	±	0.020	1.085	±	0.363
Cyperus rotundus (rhizoma)	0.0000	±	0.0000	0.0082	±	0.0010	0.0103	±	0.0078	1.020	±	0.056	1.252	±	0.206
Dimocarpus longan (arillus)	-0.0023	±	0.0012	0.0008	±	0.0006	0.0022	±	0.0015	0.905	±	0.036	0.935	±	0.108
Dioscorea oppositifolia (rhizoma)	0.0010	±	0.0017	0.0067	±	0.0055	0.0154	±	0.0100	1.365	±	0.019	0.964	±	0.059
Eleutherococcus nodiflorus (cortex)	-0.0020	±	0.0006	0.0287	±	0.0249	0.0093	±	0.0056	0.680	±	0.026	0.964	±	0.029
Ephedra sinica (herba)	0.0037	±	0.0103	0.0147	±	0.0077	0.0021	±	0.0015	1.011	±	0.113	1.050	±	0.269
Epimedium brevicornu (herba)	0.0060	±	0.0096	0.0108	±	0.0031	0.0009	±	0.0003	0.996	±	0.046	0.295	±	0.074
Eriobotrya japonica (folia)	0.0133	±	0.0069	0.0038	±	0.0313	0.0082	±	0.0057	0.994	±	0.057	1.097	±	0.309
Eucommia ulmoides (cortex)	0.0143	±	0.0078	0.0391	±	0.0281	0.0041	±	0.0010	1.030	±	0.035	1.155	±	0.224
Foeniculum vulgare (fructus)	0.0000	±	0.0000	0.0028	±	0.0006	0.0538	±	0.0381	0.844	±	0.042	0.981	±	0.014
Forsythia suspensa (fructus)	0.0120	±	0.0069	0.0003	±	0.0023	0.0051	±	0.0035	0.932	±	0.040	1.252	±	0.092
Fritillaria verticillata (bulbus)	-0.0010	±	0.0000	0.0034	±	0.0027	0.0007	±	0.0003	0.993	±	0.069	1.318	±	0.244
Gardenia jasminoides (fructus)	0.0103	±	0.0066	0.0042	±	0.0076	0.0266	±	0.0208	0.865	±	0.024	0.979	±	0.309
Gastrodia elata (rhizoma)	-0.0020	±	0.0012	0.0213	±	0.0118	nd	±	nd	0.812	±	0.087	1.338	±	0.191
Gentiana macrophylla (radix)	-0.0007	±	0.0009	0.0072	±	0.0065	0.2290	±	0.1565	0.451	±	0.081	1.054	±	0.186
Gentiana manshurica (radix)	0.0002	±	0.0001	0.0162	±	0.0060	0.0037	±	0.0026	0.943	±	0.032	1.208	±	0.449
Glehnia littoralis (radix)	-0.0027	±	0.0017	0.0311	±	0.0165	0.0847	±	0.0506	0.791	±	0.018	1.037	±	0.070
Glycyrrhiza uralensis (radix)	0.0007	±	0.0023	0.0751	±	0.0613	0.0072	±	0.0029	0.859	±	0.036	0.579	±	0.064

Houttuynia cordata (herba)	-0.0003	±	0.0012	0.0227	±	0.0142	0.0148	±	0.0114	0.534	±	0.052	0.894	±	0.069
Cryptotympana atrata (periostracum)*	-0.0010	±	0.0033	0.0200	±	0.0035	0.0012	±	0.0009	0.946	±	0.022	0.618	±	0.145
Inula helenium (radix)	-0.0030	±	0.0000	0.0009	±	0.0008	0.0543	±	0.0257	0.674	±	0.012	1.141	±	0.170
Inula japonica (flos)	-0.0007	±	0.0009	0.0037	±	0.0019	0.1378	±	0.1120	0.874	±	0.005	1.115	±	0.135
Leonurus japonicus (herba)	-0.0003	±	0.0012	0.0356	±	0.0251	0.0011	±	0.0009	0.879	±	0.028	0.228	±	0.026
Ligusticum striatum (rhizoma)	0.0000	±	0.0006	0.0166	±	0.0084	0.3190	±	0.0817	0.863	±	0.017	0.824	±	0.048
Lilium brownii (bulbus)	-0.0013	±	0.0007	0.0158	±	0.0107	0.0006	±	0.0002	0.930	±	0.093	1.096	±	0.037
Lindera aggregata (radix)	-0.0007	±	0.0003	0.0087	±	0.0069	0.0001	±	0.0001	1.021	±	0.110	0.018	±	0.010
Lonicera japonica (caulis)	-0.0010	±	0.0006	0.0330	±	0.0261	0.0005	±	0.0003	1.185	±	0.068	0.748	±	0.107
Lonicera japonica (flos)	0.0020	±	0.0021	0.0179	±	0.0078	0.0010	±	0.0005	1.171	±	0.045	0.418	±	0.115
Lycium barbarum (fructus)	0.0017	±	0.0022	0.0403	±	0.0259	0.0009	±	0.0001	0.714	±	0.061	1.086	±	0.229
Lycium chinense (cortex)	0.0003	±	0.0009	0.0030	±	0.0017	0.0028	±	0.0016	1.233	±	0.070	1.169	±	0.437
Magnolia biondii (flos)	0.0010	±	0.0012	0.0048	±	0.0029	0.0005	±	0.0001	1.244	±	0.026	0.424	±	0.118
Magnolia officinalis (cortex)	-0.0020	±	0.0015	0.0689	±	0.0353	0.0118	±	0.0082	1.606	±	0.102	0.768	±	0.241
Mentha canadensis (herba)	-0.0007	±	0.0009	0.0929	±	0.0492	0.0006	±	0.0001	1.680	±	0.173	0.691	±	0.110
Morus alba (cortex)	-0.0033	±	0.0012	0.0272	±	0.0085	0.0008	±	0.0004	1.120	±	0.029	1.339	±	0.410
Morus alba (folia)	-0.0010	±	0.0010	0.0081	±	0.0038	0.0003	±	0.0002	1.346	±	0.080	0.977	±	0.139
Nelumbo nucifera (semen)	-0.0003	±	0.0007	0.1634	±	0.1099	0.0006	±	0.0003	0.830	±	0.025	1.214	±	0.126
Notopterygium incisum (rhizoma)	0.0127	±	0.0066	0.0380	±	0.0037	0.0064	±	0.0029	0.971	±	0.045	0.695	±	0.268
Ophiopogon japonicus (radix)	-0.0033	±	0.0007	0.0535	±	0.0273	0.0001	±	0.0000	1.066	±	0.066	0.791	±	0.256
Paeonia × suffruticosa (cortex)	0.0000	±	0.0015	0.0179	±	0.0102	0.0009	±	0.0002	1.204	±	0.094	0.405	±	0.091
Paeonia lactiflora (radix)	0.0000	±	0.0010	0.0082	±	0.0067	0.0054	±	0.0025	0.866	±	0.036	0.917	±	0.070
Panax ginseng (rhizoma) (Ginseng red)	-0.0017	±	0.0015	0.2080	±	0.0243	0.1900	±	0.0993	2.345	±	0.524	0.674	±	0.121
Panax ginseng (rhizoma) (Ginseng white)	-0.0027	±	0.0023	0.4356	±	0.1599	0.1580	±	0.0689	1.296	±	0.062	0.867	±	0.059
Perilla frutescens (folia)	-0.0013	±	0.0012	0.0060	±	0.0049	0.0066	±	0.0032	0.937	±	0.122	0.870	±	0.089
Peucedanum praeruptorum (radix)	-0.0053	±	0.0017	0.0135	±	0.0049	0.0019	±	0.0011	1.210	±	0.052	1.200	±	0.282
Phellodendron chinense (cortex)	-0.0037	±	0.0029	0.0015	±	0.0012	0.0026	±	0.0013	0.945	±	0.048	1.104	±	0.281
Pinellia ternata (rhizoma)	0.0000	±	0.0000	0.0000	±	0.0002	0.0022	±	0.0014	0.881	±	0.039	0.943	±	0.086
Plantago asiatica (semen)	-0.0030	±	0.0012	0.0019	±	0.0008	0.0007	±	0.0003	0.945	±	0.050	1.208	±	0.121
Platycladus orientalis (folia)	0.0020	±	0.0025	0.0001	±	0.0044	0.0025	±	0.0012	1.067	±	0.106	1.091	±	0.116
Platycodon grandiflorus (radix)	-0.0003	±	0.0003	0.0055	±	0.0034	0.0003	±	0.0002	1.021	±	0.045	1.107	±	0.181
Pogostemon cablin (herba)	-0.0023	±	0.0019	0.0085	±	0.0058	0.0013	±	0.0008	0.991	±	0.085	0.897	±	0.162
Polygala tenuifolia (radix)	-0.0020	±	0.0010	0.0081	±	0.0066	0.0021	±	0.0009	1.072	±	0.040	1.095	±	0.274
Polygonatum kingianum (rhizoma)	0.0010	±	0.0042	0.0497	±	0.0103	0.0027	±	0.0022	0.874	±	0.027	1.222	±	0.163
Polyporus umbellatus (sclerotium)	0.0007	±	0.0007	0.0045	±	0.0021	0.0009	±	0.0007	1.014	±	0.032	0.930	±	0.107
Prunella vulgaris (spica)	0.0003	±	0.0007	0.0035	±	0.0029	0.0027	±	0.0016	1.080	±	0.064	0.832	±	0.123

Prunus persica (semen)	-0.0020	±	0.0032	0.0023	±	0.0016	0.0006	±	0.0003	1.087	±	0.102	1.008	±	0.039
Pueraria montana (radix)	-0.0007	±	0.0007	0.0076	±	0.0062	0.0026	±	0.0017	1.003	±	0.035	0.722	±	0.087
Rehmannia glutinosa (radix)	-0.0020	±	0.0012	0.0048	±	0.0025	0.0056	±	0.0026	1.116	±	0.036	0.885	±	0.087
Reynoutria multiflora (radix)	0.0000	±	0.0027	0.0416	±	0.0198	0.0031	±	0.0017	1.225	±	0.057	0.978	±	0.240
Rheum palmatum (rhizoma)	-0.0007	±	0.0023	0.0367	±	0.0300	0.0055	±	0.0039	0.971	±	0.040	0.798	±	0.109
Salvia miltiorrhiza (radix)	-0.0007	±	0.0003	0.0194	±	0.0092	0.0095	±	0.0058	0.156	±	0.048	0.611	±	0.156
Saposhnikovia divaricata (radix)	0.0003	±	0.0012	0.0067	±	0.0025	0.0006	±	0.0001	0.468	±	0.085	0.899	±	0.058
Schisandra chinensis (fructus)	-0.0030	±	0.0015	0.0006	±	0.0005	0.0002	±	0.0001	0.496	±	0.136	0.952	±	0.082
Scutellaria baicalensis (radix)	-0.0003	±	0.0015	0.1045	±	0.0620	0.0132	±	0.0057	1.927	±	0.487	0.885	±	0.073
Senna tora (semen)	0.0017	±	0.0019	0.0024	±	0.0027	0.0077	±	0.0056	1.058	±	0.047	1.167	±	0.173
Syzygium aromaticum (flos)	-0.0007	±	0.0015	0.0650	±	0.0456	0.0012	±	0.0009	1.558	±	0.063	0.683	±	0.244
Terminalia chebula (fructus)	-0.0030	±	0.0012	0.0000	±	0.0000	0.0019	±	0.0012	0.326	±	0.070	1.024	±	0.105
Tetradium ruticarpum (fructus)	0.0140	±	0.0078	0.0490	±	0.0111	0.0038	±	0.0019	1.004	±	0.009	0.457	±	0.058
Tussilago farfara (flos)	0.0147	±	0.0081	0.0216	±	0.0002	0.0037	±	0.0008	0.971	±	0.067	1.079	±	0.467
Uncaria rhynchophylla (ramus cum uncis)	0.0000	±	0.0000	0.0030	±	0.0122	0.0130	±	0.0069	0.940	±	0.012	1.019	±	0.053
Wolfiporia extensa	0.0000	±	0.0000	0.0009	±	0.0011	0.0022	±	0.0012	0.860	±	0.037	0.965	±	0.052
Zanthoxylum bungeanum (fructus)	0.0020	±	0.0010	0.0039	±	0.0128	0.0031	±	0.0015	1.001	±	0.039	0.809	±	0.006
Zingiber officinale (rhizoma, sicc.)	0.0010	±	0.0023	0.0299	±	0.0176	0.1145	±	0.0664	0.996	±	0.053	0.561	±	0.027
Zingiber officinale (rhizoma, vir.)	0.0007	±	0.0009	0.0683	±	0.0558	0.0011	±	0.0004	0.854	±	0.023	0.801	±	0.050
Ziziphus jujuba (fructus)	0.0090	±	0.0046	0.0031	±	0.0000	0.0107	±	0.0049	0.933	±	0.080	0.990	±	0.058
Ziziphus spina-christi (semen)	-0.0033	±	0.0009	0.0142	±	0.0099	0.0125	±	0.0088	0.828	±	0.035	0.922	±	0.093
Caesalpinia sappan (lignum)	-0.0007	±	0.0022	0.0326	±	0.0265	0.0005	±	0.0003	0.191	±	0.061	0.513	±	0.090

* The only non-herbal drug (Arthropoda, Insecta, Cicadidae)

Supplementary Table 3. Action of single substances on the 5-HT_{3A} and $GABA_A$ receptors

All of the substances were tested at a concentration of 1 mM with the exception of schizandrin B (100 µM)

Substance (1 mM)	Substance class	I/I_{GABA} [3 or 10 µM]			$I/I_{5\text{-}HT}$ [5 µM]		
		Mean:		SEM:	Mean:		SEM:
3,4-dihydroxybenzoic acid [1]	phenolic compound	0.616	±	0.035	0.720	±	0.260
4-hydroxy-3-methoxybenzylalcohol [1]	phenolic compound	0.976	±	0.034	0.424	±	0.083
4-hydroxybenzaldehyd	phenolic compound	1.023	±	0.071	0.571	±	0.060
4-hydroxybenzylalcohol [1]	phenolic compound	0.977	±	0.096	0.409	±	0.081
andrographolide	terpenpoid	0.224	±	0.094	0.775	±	0.030
atractylenolide III	terpenpoid	0.738	±	0.038	0.339	±	0.056
aucubin [2]	iridoid	0.810	±	0.048	0.873	±	0.079
berberine	alkaloid	0.400	±	0.049	0.424	±	0.037
boldine	alkaloid	0.136	±	0.012	0.007	±	0.016
chlorogenic acid	phenolic compound	0.893	±	0.078	0.886	±	0.060
caffeic acid	phenolic compound	0.961	±	0.031	1.240	±	0.090
eudesmol [3]	terpenpoid	not determined	±	not determined	0.099	±	0.037
ferulic acid [4]	phenolic compound	0.842	±	0.035	not determined	±	not determined
harpagoside [5]	iridoid	0.834	±	0.010	not determined	±	not determined
hesperetin	flavonoid	1.023	±	0.051	0.236	±	0.062
kaempferol	flavonoid	0.889	±	0.110	0.740	±	0.112
leonurine	alkaloid	0.129	±	0.019	0.010	±	0.020
linderane	terpenpoid	1.029	±	0.138	1.310	±	0.040
liquritgenin	flavonoid	0.721	±	0.022	0.067	±	0.025
rosmarinic acid	phenolic compound	0.487	±	0.003	0.990	±	0.070
rutin	flavonoid	0.476	±	0.044	0.227	±	0.043
schizandrin	lignan	0.419	±	0.019	0.256	±	0.052
schizandrin B (100 µM)	lignan	1.282	±	0.118	0.996	±	0.061
sclareol [6]	terpenoid	1.744	±	0.138	0.289	±	0.070
tannic acid	phenolic compound	0.070	±	0.030	0.125	±	0.023

[1] *Gastrodia elata* Blume, Orchidaceae (Jang et al., 2015)
[2] *Eucommia ulmoides* Oliv., Eucommiaceae (Viljoen et al., 2012)
[3] *Atractylodes lancea* (Thunb.) DC., Compositae (Nakai et al., 2003)
[4] *Cnidium monnieri* (L.) Cusson, Apiaceae (Hiratsuka et al., 2010)
[5] *Harpagophytum procumbens* (Burch.), Pedaliaceae (Viljoen et al., 2012)
[6] *Salvia sclarea* L., Lamiaceae (Caissard et al., 2012)

Supplementary Table 4. Calculated IC_{50} values of select ingredients on the 5-HT_{3A} and $GABA_A$ receptors

	$GABA_A$ receptor (3 μM GABA)				5-HT_{3A} receptor (5 μM 5-HT)		
**substance**	**IC_{50} ± SEM (μM)**				**IC_{50} ± SEM (μM)**		
andrographolide	66.1	±	26.8		/	±	/
boldine	111.1	±	10.5		0.53	±	0.15
leonurine	89.2	±	40.1		2.17	±	0.57
schizandrin	640	±	72.6		137	±	22.5
tannic acid	109	±	103		48.3	±	4.14

1. Supplementary Figures

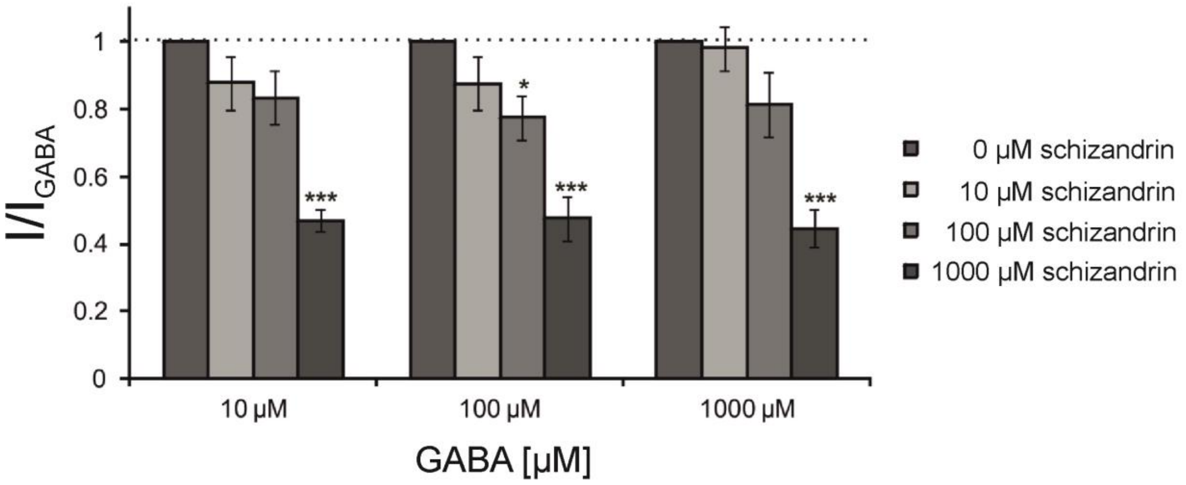

Supplementary Figure 1. **Apparent non-competitive antagonism of schizandrin on the GABA$_A$ receptor**

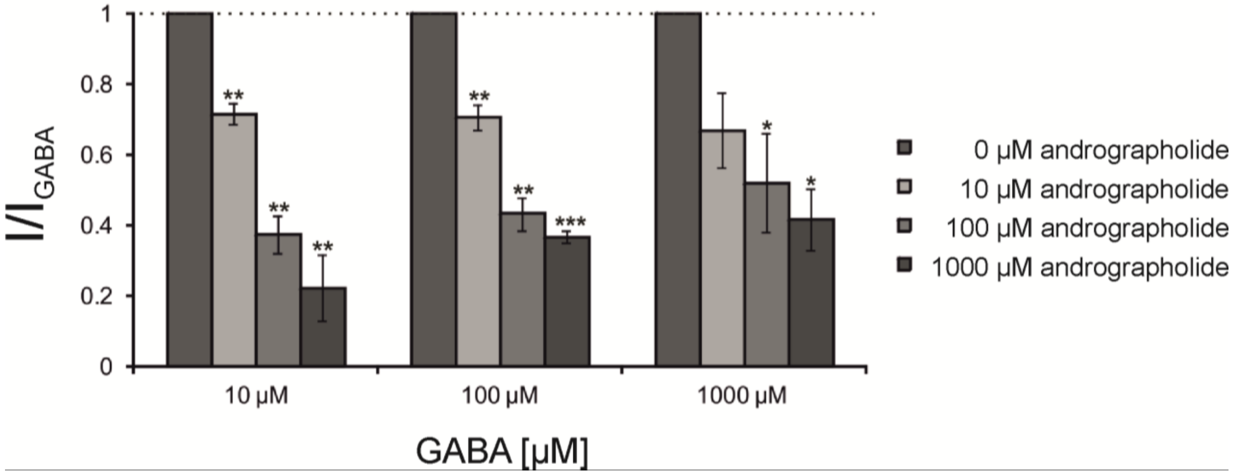

Supplementary Figure 2. **Apparent non-competitive antagonism of andrographolide on the GABA$_A$ receptor**
